# A Standardized Pipeline for Assembly and Annotation of African Swine Fever Virus Genome

**DOI:** 10.3390/v16081293

**Published:** 2024-08-13

**Authors:** Edward Spinard, Mark Dinhobl, Cassidy N. G. Erdelyan, James O’Dwyer, Jacob Fenster, Hillary Birtley, Nicolas Tesler, Sten Calvelage, Mikael Leijon, Lucilla Steinaa, Vivian O’Donnell, Sandra Blome, Armanda Bastos, Elizabeth Ramirez-Medina, Anna Lacasta, Karl Ståhl, Huaji Qiu, Dachrit Nilubol, Chandana Tennakoon, Charles Maesembe, Bonto Faburay, Aruna Ambagala, David Williams, Paolo Ribeca, Manuel V. Borca, Douglas P. Gladue

**Affiliations:** 1 U.S. Department of Agriculture, Agricultural Research Service, Foreign Animal Disease Research Unit, Plum Island Animal Disease Center (PIADC), P.O. Box 848, Greenport, NY 11944, USA; edward.spinard@usda.gov (E.S.); mark.dinhobl@usda.gov (M.D.);; 2 U.S. Department of Agriculture, Agricultural Research Service, Foreign Animal Disease Research Unit, National Bio and Agro-Defense Facility, Manhattan, KS 66502, USA; 3 CSIRO, Australian Centre for Disease Preparedness, Geelong, VIC 3220, Australia; 4 National Centre for Foreign Animal Disease, Canadian Food Inspection Agency, Winnipeg, MB R3E 3M4, Canada; 5 Oak Ridge Institute for Science and Education (ORISE), Oak Ridge, TN 37830, USA; 6 Friedrich-Loeffler-Institut, Federal Research Institute for Animal Health, Südufer 10, 17493 Greifswald-Insel Riems, Germany; 7 Department of Microbiology, Swedish Veterinary Agency, SE-751 89 Uppsala, Sweden; 8 Animal and Human Heath Program, International Livestock Research Institute, Nairobi 00100, Kenya; 9 U.S. Department of Agriculture, Animal and Plant Inspection Service, Plum Island Animal Disease Center, Greenport, NY 11944, USA; 10 Department of Veterinary Tropical Diseases, Faculty of Veterinary Science, University of Pretoria, Onderstepoort 0110, South Africa; 11 Department of Epidemiology, Surveillance and Risk assessment, Swedish Veterinary Agency, SE-751 89 Uppsala, Sweden; 12 State Key Laboratory for Animal Disease Control and Prevention, National African Swine Fever Para-Reference Laboratory, National High Containment Facilities for Animal Diseases Control and Prevention, Harbin Veterinary Research Institute, Chinese Academy of Agricultural Sciences, Harbin 100081, China; 13 Swine Viral Evolution and Vaccine Development Research Unit, Department of Veterinary Microbiology, Faculty of Veterinary Science, Chulalongkorn University, Henry Dunant Road, Pathumwan, Bangkok 10330, Thailand; 14 The Pirbright Institute, Ash Road, Pirbright, Woking GU24 0NF, UK; 15 Department of Zoology, Entomology and Fisheries Sciences, School of Biosciences, College of Natural Sciences, Makerere University, Kampala P.O. Box 7062, Uganda; 16 UK Health Security Agency, London E14 4PU, UK; 17 Biomathematics and Statistics Scotland, Edinburgh EH9 3FD, UK

**Keywords:** African swine fever virus, African swine fever, ASF, ASFV, pipeline, next-generation sequencing

## Abstract

Obtaining a complete good-quality sequence and annotation for the long double-stranded DNA genome of the African swine fever virus (ASFV) from next-generation sequencing (NGS) technology has proven difficult, despite the increasing availability of reference genome sequences and the increasing affordability of NGS. A gap analysis conducted by the global African swine fever research alliance (GARA) partners identified that a standardized, automatic pipeline for NGS analysis was urgently needed, particularly for new outbreak strains. Whilst there are several diagnostic and research labs worldwide that collect isolates of the ASFV from outbreaks, many do not have the capability to analyze, annotate, and format NGS data from outbreaks for submission to NCBI, and some publicly available ASFV genomes have missing or incorrect annotations. We developed an automated, standardized pipeline for the analysis of NGS reads that directly provides users with assemblies and annotations formatted for their submission to NCBI. This pipeline is freely available on GitHub and has been tested through the GARA partners by examining two previously sequenced ASFV genomes; this study also aimed to assess the accuracy and limitations of two strategies present within the pipeline: reference-based (Illumina reads) and de novo assembly (Illumina and Nanopore reads) strategies.

## 1. Introduction

The African swine fever (ASF) is a contagious and often deadly disease in swine caused by the African swine fever virus (ASFV), a large dsDNA virus, which originates from southern and eastern Africa. The current genotype II ASF has been causing a pandemic, which started in 2007 when the virus was introduced to the Republic of Georgia [1]. Since then, outbreaks have been reported from Europe, Asia, Oceania, western Africa, and the island of Hispaniola. While this pandemic is caused by genotype II viruses, ASF viruses of other genotypes are circulating in Sub-Saharan Africa. More recently, outbreaks of a genotype I/genotype II recombinant have been reported in China and Vietnam [2,3]. Outbreaks of ASF have very vast economic consequences in affected countries and regions, due to the disease per se, implementing control measures, and dealing with possible trade restrictions [4]. In a global economy, an accurate understanding of the outbreak strain origin and spread is important to understand the epidemiology and disease dynamics for mitigating further spread and for the development and deployment of effective vaccines.

In the event of an outbreak, the rapid identification of the virus genotype/biotype is readily achieved using partial [5] or full length p72 protein [6] characterization, and depending on the laboratory capabilities, either by full length genomic sequencing allowing for gene-by-gene-biotyping [7], or by multi-locus sequence analysis [8,9,10,11]. While partial sequencing may be an important first step in virus typing, full genome sequencing remains the most informative and comprehensive means of gaining valuable insights into outbreaks. However, full genome sequencing requires significant infrastructure and bioinformatics expertise to assemble reads into consensus sequences, annotate genomes, and accomplish other routine analysis requirements that are out of reach for many countries in endemic settings. Often, diagnostic or research labs must dedicate resources to develop their own method of NGS analysis, or use a generalized viral analysis or annotation pipeline, which may underperform for specific viral species.

Numerous features and regions within the ASFV genome present challenges when its assembly from NGS reads is attempted. The ASFV has a large double-stranded DNA genome, which varies in length from 170 to 195 kb [7]. The genome can be broadly characterized into five sections: a central conserved region (125 kb) flanked by two variable regions (38–47 kb and 13–39 kb) and two terminal inverted repeat (ITR) regions located at the 5′ and 3′ ends of the genome [12]. The absence of directed PCR amplification or targeted viral enrichment (e.g., MyBaits) typically leads to a minimal number of reads originating from the ASFV in comparison to the host, resulting in a limited depth of coverage [13,14,15,16]. Overall, a consensus sequence can be hard to reconstruct from de novo assembly due to both the relative length of the ASFV genome and an inadequate number of sequenced ASFV reads. Furthermore, the success of directed PCR amplification is dependent on primer sets that are able to amplify specific regions across the entire genome, general enough to cope with multiple genome variants, and sufficiently flexible to account for the possibility of large deletions and duplication/translocation events [17,18]. In addition to the challenges posed by its length, the assembly of an ASFV genome is further complicated by the presence of several low complexity regions [7]. Complementary ITR regions that vary in length between different genomes are present at both the 5′ and 3′ end of the genome and are hard to resolve with short reads [18]. In addition, the genome contains multiple homopolymer runs where it is difficult to discern sequencing errors from true genomic variants or mixed populations of virus isolates [19]. The annotation of the ASFV proves to be challenging because its genome has the potential to encode up to 190 open reading frames (ORFs) [20]. The names of most ORFs have been standardized according to the nomenclature established by Rodriguez et al., where ORFs were named according to the letter of the EcoRl fragment, the number of amino acids encoded, and the orientation of the gene (left or right, denoted as L or R) [21]. Still, the analysis and annotation of ORFs becomes more complex due to some genes having multiple names (e.g., UK (DP96R) and NL (DP71L)) and some genomes having gene duplicates (I10L) [22]. Additionally, the ASFV is responsible for encoding multiple proteins falling under five distinct multigene families (MGF) named MGF 100, MGF 110, MGF 300, MGF 360, and MGF 505, that are encoded within the 5′ and 3′ variable regions. These families are categorized based on conserved motifs and on the average amino acid length of the genes they contain. Homopolymeric stretches are commonly present in the coding sequences of these genes and may lead to gene loss, truncation, or fragmentation, thus complicating ASFV annotation.

Different analysis methods have the potential to cause problems to the uniformity of the ASFV assembly and downstream analysis, classification, and deep epidemiological investigations. Further, manually performing every step of the assembly process (read filtering, assembly, annotation, file formatting, and analysis) is time-consuming and labor intensive, which can cause delays in identifying new ASFV variants. Further, many labs lack the computational infrastructure and the personnel with extensive bioinformatic experience needed to process NGS data.

To address these concerns, we developed a pair of related “Raw-Reads-to-Near-Publication” pipelines conveniently housed within a single docker/singularity container. This approach streamlines the installation process, standardizes the computational environment, resolves potential library and dependency compatibility issues, and hosts all required reference sequences. For installation instructions, see Section 2.2. The pipeline is automated based on the raw reads. The first pipeline only requires an input of paired Illumina reads which are first processed for quality control and then de novo assembled into contigs. In general, it is hard to accomplish the assembly of a complete genome solely with Illumina reads. Accordingly, the de novo assembled contigs are used to predict a closely related reference genome to which the processed reads are mapped, and a consensus sequence is extracted. The second pipeline requires both Illumina and long Nanopore reads and provides accurate results without the need for a reference genome. After processing the Illumina reads, when the input quality is sufficient both the Nanopore and Illumina reads are de novo assembled and a single contig representing the complete genome is extracted from the assembly. In cases with a lower input sequence quality, multiple smaller contigs would produce outputs representing partial ASFV sequences. Post assembly, both pipelines generate mapping coverage and quality graphs, annotate ORFs and low-quality regions of the consensus sequence, determine the genotype and likely biotype of the consensus, and create a summary text file. Further, to expedite the process of submitting the genome to NCBI, the reference sequence includes all user provided metadata in the FASTA header and the annotations are conveniently processed into NCBI’s five-column, tab-delimited feature table format. To prolong its life and usefulness, the code is entirely public and open source. Here, we demonstrate the use of an automatic pipeline capable of analyzing and annotating ASFV genomes from NGS data. While no automated pipeline is perfect and a human verification is essential, this pipeline can save time and resources for the ASFV research community. Whenever possible, it is strongly advised to sequence novel genomes using both Illumina and Nanopore reads in order to achieve optimal results. Still, in cases where only Illumina reads are accessible, a thorough evaluation of the final assembly in comparison to the reference is crucial and highly recommended.

## 2. Materials and Methods

### 2.1. Pipeline Overview

**Figure 1:**
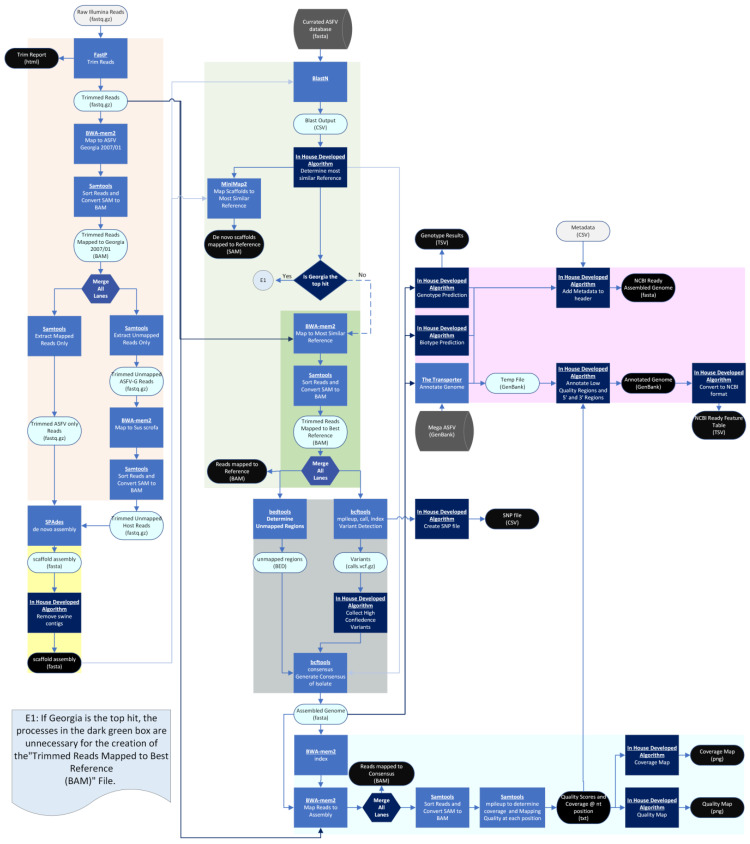
Workflow of the reference-based pipeline. User provided input files (grey oval), final output files (black oval), temporary files (light blue oval), software (blue box), in house developed algorithms (dark blue box), and databases (dark grey cylinders) are represented by the indicated shapes and colors. The generalized processes of read correction and filtering (orange), de novo assembly (yellow), reference prediction and mapping (light and dark green), error correction (grey), coverage and quality determination (light cyan), and genome characterization (pink) are boxed by the indicated colors.

**Figure 2:**
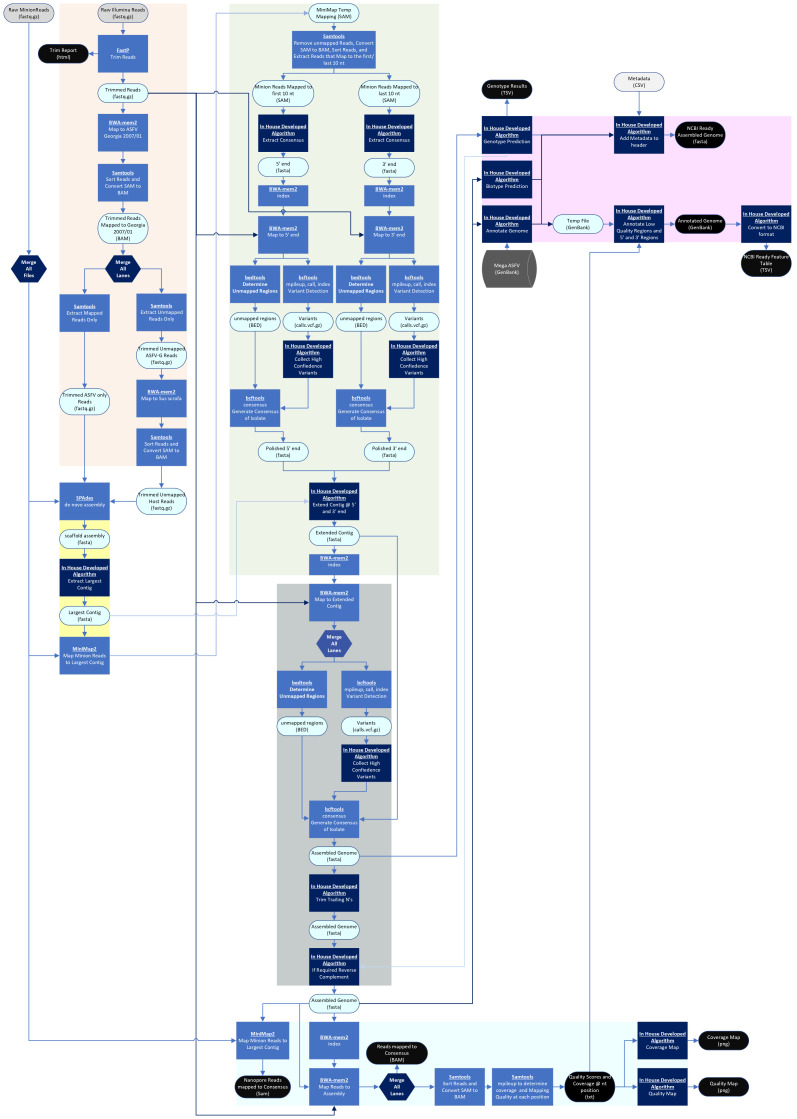
Workflow of the de novo assembly pipeline. User provided input files (grey oval), final output files (black oval), temporary files (light blue oval), software (blue box), in house developed algorithms (dark blue box), and databases (dark grey cylinders) are represented by the indicated shapes and colors. The generalized processes of read correction and filtering (orange), de novo assembly (yellow), contig extension (light green), error correction (grey), coverage and quality determination (light cyan), and genome characterization (pink) are boxed by the indicated colors.

The pipeline developed in this report is split into two different pipelines that share several steps: reference-based (Illumina paired-reads only) (Figure 1) and de novo assembly (Illumina and Nanopore reads) pipelines (Figure 2). The inclusion of a Nanopore directory dictates the choice of pipeline. All Linux commands that call one of the several packaged freeware Linux programs can be found in Supplementary Figure S1.

#### 2.1.1. Illumina Reads QC (Both Pipelines)

The QC processing of Illumina reads is the first step in both pipelines (Figure 1 and Figure 2, orange box). Up to four sets of Illumina reads may be submitted. Reads may be generated from any source material (e.g., tissue culture, host issue, and amplicons). As batch effects may be present in reads generated in different lanes or from different runs, all paired-read sets are processed individually prior to mapping or assembly. First, paired-reads are trimmed with FastP [23,24] using a provided database of all Illumina adapters, and are processed with the following options: ambiguous nucleotides (max = 2), minimum length (50 nt), minimum quality (min phred score = 20), and nucleotide composition (25 and 5 nt are removed from the 5′ and 3′ ends, respectively). Only paired-reads are retained. As indicated in Figure 1 and Figure 2 as “Trim Report”, the results of FastP are saved as a user-friendly html file. Users should ensure there are no notable differences between reads generated on different lanes during the same Illumina run. Next, to identify which reads are likely from the ASFV and to remove the contaminating host sequence, the trimmed reads are first mapped against the ASFV Georgia 2007/1 (FR682468) [1] genome using the default parameters of BWA-MEM2 mem [25]. Samtools (version 1.20) [26] is then used to sort, extract, and save the reads that properly aligned and stayed as pairs using the flag “view -f 0x2”. During this step, reads that do not map to the ASFV Georgia 2007/1 reference genome are collected for recovery. Failed mappings occur when reads have a high sequence variation and/or large insertions relative to the ASFV Georgia 2007/1 genome. Accordingly, to rescue such reads, the following steps are performed: (1) reads that do not map to ASFV Georgia 2007/1 are sorted, extracted, and saved using Samtools [26] with the flag “view -f 0x4”. (2) These paired-reads are subsequently mapped to the *Sus scrofa* reference genome [27] using BWA-MEM2 mem [25]—to reduce the likelihood of the accidental mapping of ASFV reads, the minimal seed length can be adjusted to 45 (-k = 45) from the default value of 19; and finally, (3) to remove contaminating host reads, paired-reads that do not map to the *Sus scrofa* genome are sorted, extracted, and saved using Samtools [26] with the flag “view -f 0x4”. Again, paired-reads that fail to map to *Sus scrofa* are kept, since they are more likely to be reads originating from the ASFV.

#### 2.1.2. De Novo Assembly (Both Pipelines)

The de novo assembly portion of the pipeline is outlined by the yellow box in both Figure 1 and Figure 2. This is the first section in which the pipelines differ. In both pipelines, trimmed Illumina paired-reads that mapped to ASFV Georgia 2007/1 and trimmed Illumina paired-reads that failed to map to either ASFV Georgia 2007/1 or the *Sus scrofa* genome are de novo assembled using SPAdes v 3.15.4 [28] with the careful flag set and kmer sizes set to 21, 33, 55, 75, and 99. In the de novo assembly pipeline, Nanopore reads are included as a SPAdes input and the Nanopore option of SPAdes is set. SPAdes produces a scaffold FASTA file which contains multiple assembled contigs [24]. In the reference-based pipeline, the scaffolds are compared against the *Sus scrofa* genome using blastn [29] with the following parameters: -evalue = 0.001, -max_hsps = 1, and -max_target_seqs = 1. Any contigs that match are removed from the assembly. This scaffold file is saved as “ProjectID_scaffold.FASTA” and the user may analyze this file as it may contain a completely assembled genome. Following assembly in the de novo assembly pipeline, the largest contig is extracted from the scaffold file and analyzed for length. As a proxy for checks on the read sequence quality and coverage, if the largest contig is smaller than 150,000 nt, the pipeline is stopped, and an error message is given. Typically, this occurs when certain regions could not be sequenced with Illumina technology.

#### 2.1.3. Reference Prediction and Mapping (Illumina Only)

In the reference-based pipeline (Figure 1, green box), to find the best matched reference genome available, all contigs larger than 1250 nt from the host-free scaffold assembly are compared against a curated ASFV database representing all currently classified ASFV genomes using the following parameters: -evalue = 0.001 and -max_hsps = 1. The reference database was created to include a diverse set of ASFV genomes (Table 1). The accumulative bitscore for each reference is calculated to determine the most similar references. The host-free scaffolds are then mapped to the most similar reference genome using the asm5 preset of Minimap2 [30,31]. No additional processing or analysis is conducted on this generated read-alignment file. Nonetheless, this mapping is included in the final output as a SAM file as it may be necessary for the user to compare the de novo assembly with the reference in order to identify any potential large genomic deletions, insertions, or errors compared to the reference.

Following the identification of the most similar ASFV reference genome, the trimmed Illumina reads are mapped against it using the default parameters of BWA-MEM2 mem [25] and sorted using Samtools [26] (Figure 1, dark green box). This mapping file is included in the final output as a BAM file, and it is recommended that the user view this file using their favorite read-alignment software. It is important to note that this file will also contain any unmapped reads. As explained in the Figure 1 box labeled E1, there is no need to repeat this process if ASFV Georgia 2007/1 was determined to be the best match, as the “mapped reads to reference” BAM file was already created during the QC processing of the Illumina reads in the yellow box of Figure 1.

#### 2.1.4. Contig Extension (De Novo Only)

Despite the inclusion of long Nanopore reads in the de novo assembly, the resolution of the ITR regions that exist at the 5’ and 3’ ends of the ASFV genome can be challenging for de novo assemblers. Further, our analysis of Nanopore reads that map into these regions terminate at different lengths, possibly indicating that these regions may be of variable length even within a viral population. As a result, the largest assembled contig often lacks these two regions of the genome. Accordingly, we developed the following methodology to extend the largest contig (Figure 2, green box). First, the Nanopore reads are mapped to the largest de novo assembled contig using the map-ont preset of Minimap2 [30,31]. Unmapped reads are removed by using the Samtools view function with the following flag: “-F 4” [26]. Next, the sequence alignment file is sorted using the Samtools sort function and indexed using the Samtools index function [26]. Nanopore reads that map to the first 10 nucleotides or last 10 nucleotides of the contig may contain a substantial sequence length which is unaligned and flanks the constructed contig. Determining the consensus of these unaligned sequences can be used to extend the contig. To isolate these reads, the Samtools view function [26] is used to create two sequence alignment files: The first sequence alignment file only contains reads that align to at least the first 10 nucleotides of the contig. The second sequence alignment file consists of reads that only align to at least the last 10 nucleotides of the contig. Next, we use an in house developed python script to convert the sequence alignment files into data frames. Unaligned sequences that are flanking the contig and 1000–10,000 nt long are extracted and sorted using a bin size of 500 (e.g., 1000–1499, 1500–1999). Bins that have less than 12 sequences are removed from further analysis. Sequences within the bin that contains the set of longest sequences are extracted to a FASTA file. It is noteworthy that to save on computational time, only a maximum of 100 sequences will be extracted. These extracted sequences are then aligned using the default parameters of MUSCLE [32], and subsequently, the consensus sequence is derived using the function gap_consensus from the python library BioPython’s module Bio.Align.AlignInfo.SummaryInfo with the following parameters: threshold = 0.2, ambiguous = ‘N’, and require_multiple = 1 [33]. Due to the low sequencing quality inherit to Nanopore reads, the consensus flanking sequences must be corrected with the trimmed Illumina reads. This is performed by first indexing both consensus flanking sequences via the Samtools index [26], and then mapping the trimmed Illumina reads using the default parameters of BWA-MEM2 mem [25]. Variants are detected using the default parameters of bcftools mpileup, except the max depth is changed to 100. Variants are then called using the bcftools call with the multiallelic-caller and variant-only options enabled [26]. Variants that are represented in >50% of the Illumina reads are retained using a custom python script. Unmapped regions are determined using bedtools genomecov [34,35]. Using the variant and unmapped region files, the bcftools consensus is used to extract the corrected flanking consensus sequences [26]. Both the 5′ flanking consensus sequence and the 3′ flanking consensus sequence are then appended to the largest contig assembled from SPAdes. Finally, the trimmed Illumina reads are mapped back to the extended contig using the default parameters of BWA-MEM2 mem and the pipeline continues with error corrections [25].

#### 2.1.5. Error Correction/Variant Identification and Consensus Extraction (Both Pipelines)

After reads are mapped to either the de novo assembled and extended contig (de novo assembly pipeline) or the best reference (reference-based pipeline) error correction (de novo assembly pipeline) or variant detection (reference-based pipeline), the identification of unmapped regions (both pipelines) must be performed. While fundamentally different, functionally, the process of variant detection and error correction are the same, and both pipelines share the same initial steps. The default parameters of bcftools mpileup, with the exception that the max depth for indel and SNP identification is changed to 10,000 and bcftools call, with the multiallelic-caller and variant-only options enabled, are used to identify and call variants/errors [26]. Variants that are represented in >50% of the Illumina reads are retained using a custom python script. For the reference-based pipeline, these results are included in the final output. Unmapped regions are determined using bedtools genomecov [35]. Using the variant and unmapped region files, the bcftools consensus is used to create the corrected consensus sequence [26]. In the de novo assembly pipeline, this consensus sequence is further corrected. First, any ambiguous nucleotides at the 5′ or 3′ end of the genome are trimmed using BioPython’s module Bio.Seq [33] and a custom python script. Next, when visualizing an ASFV genome, *B646L* has historically been oriented on the reverse strand. Accordingly, the orientation of *B646L* is determined using a custom python script and blastx [29] with the following parameters: max_hsps = 1, max_target_seqs = 43, and evalue = 0.001 [29]. If *B646L* is oriented in the wrong direction, then the entire genome is reverse complemented.

#### 2.1.6. Developing Statistics and Coverage/Quality Map Images (Both Pipelines)

In both pipelines, the consensus genome is indexed using the BWA-MEM2 index and the trimmed reads are mapped to the consensus using the default parameters of BWA-MEM2 mem [25] (Figure 1 and Figure 2, light cyan box). This read-alignment file is provided in the final output as a BAM file. Next, unmapped reads are removed using the Samtools view function with the “f 0x2” flag, and the resulting read-alignment file is sorted using the Samtools sort function [26]. The Samtools mpileup function [26] is used to determine the coverage and mapping quality at each position using the following parameters: the max depth of cover is infinite, output mapping qualities are encoded as ASCII characters, and all positions are included. The statistical analysis is provided in the final output as a simple tab-delimited text file that is then used to create graphs that display the read-mapping quality and depth of coverage. Graphs are provided in the final output as PNG image files. In addition, in the de novo assembly pipeline, Nanopore reads are mapped to the consensus sequence using the map-ont preset of Minimap2 [30,31]. The read-map alignment is provided as a SAM file, but no further analysis is performed using this file.

#### 2.1.7. Biotyping, Genotyping, and Annotation (Both Pipelines)

In both pipelines, the consensus genome is also fed into a P72-based genotyping tool and a gene-by-gene biotyping tool [36]. If the consensus sequence is of a low quality, which results in insertions/deletions (indels) to the P72 coding gene (*B646L*), a warning message is provided. The biotype [7] and genotype [6], along with the user provided metadata, are added to the header to create the final consensus sequence’s FASTA file. This FASTA file is formatted to streamline the NCBI genome submission process, eliminating the need for manual metadata input. To annotate the genome and create a GenBank file, a script known as The Transporter [37] is utilized. It leverages as input a curated database of over 2000 ASFV protein coding sequences, both in DNA and peptide form, and for each of them tries to find the best match in the newly assembled genome by combining several BLAST-based methods (blastn, blastp, and tblastn). The best match for each set of overlapping matches at each location in the genome is then selected and exported to the GenBank file. Subsequently, the statistical analysis that was previously performed is used to indicate the ITR regions and areas with a low read-mapping quality in the final GenBank outfile. Lastly, all annotations present in the generated GenBank outfile are converted into NCBI’s five-column, tab-delimited feature table format, which can be directly uploaded during the NCBI genome submission process.

### 2.2. Usage

The most recent version of the program and instruction for installation and usage can be found on GitHub https://github.com/Global-ASFV-Research-Alliance/ASFV_Pipeline/tree/main (accessed on 10 May 2024). There, the instructions from installation to usage are provided for both docker and singularity.

### 2.3. Interpretation of Outputs

Several outputs are provided with this pipeline. The most important files are the summary file, the feature table, the GenBank file, and the consensus genome.

The summary file includes descriptions and information about all other files within the process, as well as the genotype and biotype. Additionally, it includes the references to the programs and software packages used.

The feature table and consensus file are NCBI ready, although they should be checked for any flaws. The easiest way to check this is to look at the processed GenBank file for any strange annotations, conserved genes in the incorrect location, genes that contain the note “poor mapping quality in this region”, and by examining the various read-alignment files. Within the GenBank file, each annotated gene has a ‘best reference match’. When interpreting this result, it is important to note that multiple reference genomes can have the same amino acid sequence for the product of a particular gene, and this is not an exhaustive declaration of all reference genomes that encode this sequence.

## 3. Results and Discussion

### 3.1. Filtering the Illumina Reads for Host Sequence Does Not Result in a Large Loss of Reads

**Figure 3:**
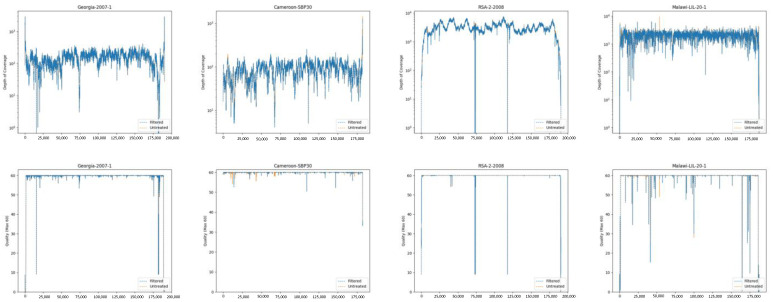
Coverage (top row) and mapping quality (bottom row) of trimmed Illumina reads that have (blue line) or have not (orange line) been processed to eliminate host reads as described in the Materials and Methods section.

To ensure that there was no significant loss of ASFV reads during the step that discards host reads, Illumina reads that were generated from a diverse set of genomes that share a 92% to 97% average nucleotide identity to ASFV Georgia 2007/1 were analyzed. All reads were trimmed according to the procedure described in the Materials and Methods section. Subsequently, the reads were aligned to their corresponding reference genome, extracted, and quantified, or they were subjected to the host-filtering process to remove pig genomic sequences, as outlined in the Materials and Methods section, before being mapped to the reference genome, extracted, and quantified. Not surprisingly, a large percentage of reads (96 to 97%) generated from samples that had not undergone an enrichment step (all samples except RSA/2/2008 [38]) were of sus scrofa origin (Table 2). The host-filtration step did not result in a noteworthy loss of paired-reads of ASFV origin (0.00 to 0.97%), a decrease in average coverage, or a loss of coverage uniformity and quality (Figure 3), thus validating our methodology.

### 3.2. De Novo Assembly ASFV DR-1980

ASFV DR-1980 is a genotype I, biotype 1 strain isolated from the Dominican Republic during their initial outbreak of the ASFV in the early 1980s. Originally, the genome was assembled in the CLC Genomics Workbench (Qiagen, Hilden, Germany) (v21) from 377 Minion reads and 17 million ASFV paired-end Illumina reads using the “De Novo Assemble Long Reads and Polish with Short Reads” workflow, resulting in a few contigs; however, consolidation to a single contig required additional manual steps [40]. To test our pipeline, we submitted the original raw Illumina and Nanopore reads into our pipeline, which resulted in the assembly of a single 181,907 nt contig that had an average coverage of 8915 Illumina paired-reads. It contained a single unresolved region made of a run of 97 ambiguous nucleotides that was easily manually resolved using the Nanopore read-alignment file. This region was evident by examining the read-mapping graph and was annotated and flagged as having poor mapping quality in the GenBank file. Compared to the CLC Genomics assembled genome, our de novo pipeline assembled genome was 893 nt shorter at the 5′ ITR, 919 shorter at 3′ ITR, and contained six variants. One variant was a 25 nt deletion within the 3′ ITR that was confirmed to be the consensus sequence by 20 of the 26 Nanopore reads. The remaining five variants cannot be confirmed or dismissed and are examined further in Section 3.3. In summary, the pipeline was able to slightly improve upon the DR-1980 assembly that was assembled with the CLC Genomics Workbench. Moreover, a significant amount of time was saved by automating the annotation.

### 3.3. Reference-Based Assembly of DR-1980

During the reference-based workflow, Illumina reads are aligned to a closely related strain and a consensus sequence is generated. The success of the procedure depends on the similarity between the reference genome and the newly sequenced isolate. This can be challenging, as some historic or exotic ASFV isolates may not closely match the genomes in the database. Additionally, significant insertions or deletions may be found in the newly sequenced genome compared to the reference strain. Still, if the sequencing quality is high and provides sufficient coverage across the newly sequenced genome, these large indels should be apparent by mapping the contigs generated from the de novo assembly step present within the reference-based pipeline to the reference genome. To test the referenced-based workflow, only the Illumina reads sequenced from DR-1980 were submitted to the pipeline. The outputs generated from the de novo assembly (Illumina only) and from the mapping against a reference were compared to the de novo assembly (Illumina and Nanopore). The two largest contigs assembled via the de novo assembly were 168,413 and 14,284 nt, respectively. An overlap of 89 nucleotides was observed between the two contigs, with 86 nucleotides displaying homology. Nevertheless, the presence of three discrepancies prevented the consolidation of these two contigs into a larger single contig. Not surprisingly, the reference-based pipeline predicted L60, a genotype 1 strain collected during the initial phase of the outbreak that eventually reached the Dominican Republic, to be the most similar ASFV reference genome. The corrected consensus sequence was 182,348 nt long and had 11 variants compared to the de novo assembled (Illumina and Nanopore) DR-1980 genome. One of these variants was an 87 nt deletion within the coding region of EP402R that can clearly be defined as an error resulting from the reference-based procedure, as this region was correctly assembled in the de novo assembly both with and without the support of Nanopore reads. The presence of this error underscores the necessity of conducting a manual examination of the reference-based outputs, as larger insertions that deviate from the reference may be absent from the final consensus sequence. Five more of these variants of less significance can be classified as errors, as they were not present in any de novo assembly (via the pipeline with and without Nanopore and CLC Genomics). Three can be classified as indels within homopolymeric runs of Cs or Gs, which were located within the ORF of MGF_360-13L, the ORF of hypothetical protein ACD_00320, and at the 3′ ITR region. The last two discernable differences were two dinucleotide substitutions that occurred at the 3′ ITR outside of any ORF. The remaining five variants cannot be confirmed or dismissed and are examined further in Section 3.3. The errors that resulted from the reference-based workflow compared to the de novo assembly, with the support of Nanopore reads, highlight the advantages of the de novo assembly. Still, if the technology is unavailable, reference-based processing can produce a satisfactory final genome, as long as a detailed manual analysis of the output is performed.

### 3.4. Shared Unresolvable Variants DR-1980

Each position in the consensus sequence created in both the de novo and reference-based pipelines reflects the most frequently occurring nucleotide at that specific position within the aligned reads. During the assembly of DR-1980, we detected the existence of five regions where two equally probable variants were observed. Four minor variations were observed in homopolymeric stretches of Gs (10 to 12 nt) that were located entirely within intergenetic regions. Both the CLC assembled genome and reference-based consensus, as compared to the genomes assembled by the de novo pipeline (with or without Nanopore reads), differed by an addition or loss of a single G. In all cases, the analysis of each assembly’s Illumina read-alignment file indicated that the correct number of Gs was represented. Nanopore reads could not be used to validate these regions as Nanopore sequencers do not accurately sequence homopolymeric or low-complexity regions. The last variant was a 36 nt sequence within the central variable region (CVR) of B602L. Coded within the CVR are tandem repeat sequences, with the number and composition differing between strains. This sequence was present in both the genome assembled by CLC and in the consensus produced by the reference-based pipeline, but it was absent from the genomes assembled by the de novo pipeline (with or without Nanopore reads). It should be noted that this ORF is indicated as having a poor mapping quality in the GenBank file created by the de novo assembly pipeline. Again, the analysis of each assembly’s Illumina read-alignment indicated that the correct sequence was represented, highlighting the potential uncertainty that occurs within extremely low-complexity regions that are sequenced and assembled with any technique.

### 3.5. De Novo Assembly ASFV Ghana2022-35, a Less Than Perfect Isolate

ASFV Ghana2022-35 is a genotype II, biotype 2 strain that was isolated from a domestic pig in Ghana in 2022 [41]. Initially, the genome was de novo assembled from 249,624 Illumina and 131,918 Nanopore reads using the default settings of the “De Novo Assembly Long Reads and Polish with Short Reads” workflow in the CLC Genomics Workbench (v21) (QIAGEN). The consensus sequence was corrected by aligning reads to the assembled genome and annotated by analyzing all ORFs predicted by the CLC Genomics Workbench against ASFV Georgia 2007/1 using tblastn [29]. From our original assembly, the analysis of the Illumina read-alignment file revealed that certain regions of the Ghana2022-35 genome had not been sequenced. Accordingly, we wanted to determine how a genome with a poorly uniformed sequencing coverage would perform in our de novo assembly. These same untreated Nanopore and Illumina reads were fed into our pipeline, resulting in a single contig. Present within the assembled contig were two ambiguous regions composed of a string of Ns. These regions were evidently of a low quality in the read-mapping graph. As there were no Illumina reads that spanned this region, these regions failed to automatically resolve to a consensus sequence. While these two regions were easily manually corrected by examining the Nanopore-alignment file, these results highlight the need for high quality Illumina reads to cover the entire genome. It should be noted that, for samples where the sequencing quality is significantly low, leading to the formation of a small contig (<150,000 nt) or indels in the conserved B646L, an error is logged to the metadatastorage.csv file and the user is notified. Compared to the CLC Genomics assembly (184,713 nt), the genome assembled by the pipeline was slightly shorter (183,872 nt). This difference can be attributed to the reduction of 485 nt at the 5′ ITR and 356 nt at the 3′ ITR, though both ends of the genome still contained the DP60R ORF. The genome that was de novo assembled by the pipeline had six variants compared to the genome assembled via CLC Genomics. All variants were simple single or dinucleotide indels or substitutions that were found within the 5′ or 3′ ITR regions. Again, like the DR-1980 assembly, read-alignments confirm these variants for either assembly, highlighting the fluctuations that can occur within low-complexity regions and that are inherent to different assembly methods. According to our analysis, our pipeline was able to adequately assemble the Ghana2022-35 genome with minimal manual correction.

### 3.6. Reference-Based Assembly Should Only Be Used for Highly Similar Genomes

In western Africa, there are two distinct variants of ASFV Georgia 2007/1 that have caused outbreaks since at least 2020. Typified by Ghana2022-35 and Nigeria-RV502, both variants have a 6500 nt deletion within the 5′ variable region that results in a loss of 14 genes. The two strains differ at the 3′ region. Ghana2022-35 is similar to ASFV Georgia 2007/1 in that the 3′ ITR is flanked upstream by MGF_360-21R within the 3′ variable region. Nigeria-RV502 contains a reverse complement duplication of a region composed of the 5′ ITR, the MGF_360-1L ORF and, partially, the MGF_360-2L ORF that recombined within the MGF_360-21R ORF. This creates a fusion between MGF_360-21R and MGF_360-2L and duplicates the MGF_360-1L ORF to the 3′ end of the genome. Due to the high degree of similarity in the left variable region and the conserved central region of the Ghana2022-35 and Nigeria-RV502 genomes, during our initial evaluation of the reference-based pipeline, we discovered challenges in accurately determining the reference genome for sequences derived from Ghana2022-35 and Nigeria-RV502, particularly when dealing with low-quality sequences. Consequently, we opted to exclusively incorporate Nigeria-RV502 in our database of reference genomes, as it is more straightforward to detect deletions in a genome than insertions. To test the referenced-based workflow, only the Illumina reads sequenced from Ghana2022-35 were submitted to the pipeline. Again, since this genome has regions that were not sequenced correctly in the Illumina reads, it was not surprising to see that the initial de novo assembly within the reference-based pipeline produced four contigs that were 168,608, 11,520, and 1174 nts long. The largest contig encompasses half the 5′ variable region, the conserved central region, the 3′ variable region, and the 3′ ITR. As expected, the examination of this contig indicates that the reverse duplication of the 5′ region to the 3′ is not present. Furthermore, in addition to the genome being split into several contigs, one could notice a lone variant, namely a single SNP situated within the 5′ ITR, from comparison with the de novo assembly that incorporated Nanopore reads. Since Ghana2022-35 was not included as a reference, the pipeline accurately identified Nigeria-RV502 (OP672342) as the best match reference and a consensus sequence was generated based on this read-alignment. The consensus sequence generated from this read-alignment erroneously had a duplication event; however, the examination of the mapping quality graph indicates that this region is problematic, as it has a mapping quality of zero. Furthermore, as indicated, examining the contigs generated by the Illumina-only de novo assembly after mapping them to Nigeria-RV502 reveals a large unaligned end where the duplication/translocation event occurs. Thus, it is important to understand the limitations of an Illumina-only assembly and thoroughly examine the outputs. These findings imply that the use of reference-based workflows should be limited to genomes that exhibit minimal deviations when compared to a well-established reference. However, in cases where a reference-based assembly is the sole option, an analysis of the assembled contigs aligned with the reference genome can provide insights into the presence of substantial deletions or insertions with respect to the consensus genome.

## 4. Discussion

The pipeline presented here to reconstruct ASFV genomes from sequencing data standardizes the way ASFV sequencing reads from Illumina and Nanopore technologies can be processed with either alignment to the closest reference or de novo sequencing. It allows for automatic annotation. This approach aids researchers to contribute to public databases by providing them with a ready-to-upload annotation file. We were unable to obtain any publicly available PacBio NGS reads for ASFV; however, because the reads can be exported in the same format as Nanopore, these reads can be used with SPAdes and minimap2, and since PacBio has a reported higher accuracy, we believe that PacBio reads could be substituted for Nanopore reads in this pipeline. Further testing using this pipeline is required to confirm this.

Here, we show that our automated pipeline can significantly cut down on time and resources for ASFV genome assembling and annotation. Although no automated pipeline is perfect, with the addition of minor human verification around problem regions, highly accurate genomes are possible with minimal effort. For optimal results, it is strongly advised to sequence novel genomes using both Illumina and Nanopore reads.

As NGS sequencers and base calling improve, it might be possible to standardize a de novo pipeline that only uses long-read technology. However, at the time of this report and available datasets, Nanopore reads were not sufficient to perform good-quality de novo sequencing of the ASFV. As the sequencing depth needed for quality Nanopore assemblies has not been established, and would require extensive testing, we were not comfortable including a Nanopore-only pipeline in the current build of the pipeline. It is also possible that, as new emerging ASFV isolates are discovered, minor modifications to the pipeline might be needed. That is why we uploaded it to open-source repositories, where it can be picked up and suitable changes can be made by us or other groups in the ASFV community.

A recognized benchmark for the sequencing depth in specific segments of the ASFV genome could aid in sequencing investigations and potentially enhance the accuracy of the consensus. Additionally, the standard could be implemented to determine if various regions of the genome exhibit varying coverages during a specific sequencing run. While low coverage in some regions happens consistently—for example, coverage in ASFV 8DR (EP402R) encoding CD2 typically has low coverage than elsewhere in the genome when using Illumina technology—most of the dips in coverage on any particular sequencing run tend to be variable. The reason for the variability is unknown but is likely due to small differences in the procedures used during DNA extraction or library preparation. Once a standard is set in the future, it could be incorporated into the pipeline and adapted to assign a score to each nucleotide based on its coverage and the position of the genome and expected levels of variability, resulting in quality information being added to the reconstruction of newly sequenced isolates.

In general, as very few ASFV genomes have been sequenced outside of the current pandemic strains, we think that more sequences of near-complete ASFV isolates would be of value even when they are not perfect or when it is not possible to perform more sequencing runs to close coverage gaps. This is illustrated by our results from historical isolates in Cameroon; in cases where the isolates are not available for re-sequencing [39], the publication of near-complete genomes can still provide valuable insights into the variation and diversity of the ASFV genome.

## 5. Conclusions

In conclusion, this newly designed pipeline for the analysis of ASFV sequencing data provides an accurate and standardized methodology as a platform that is simple to use and freely available and enables researchers to generate high quality genome sequences for the ASFV. Our evaluation of the pipeline has demonstrated that the best practice, in particular when sequencing isolates of the ASFV in new outbreak areas, is to use both Illumina and Nanopore NGS reads, which is in agreement with other ASFV NGS analysis approaches [18]. Although it is possible to obtain high quality genomes from Illumina reads alone, some sequencing analysis may require additional manual validation for complete accuracy. Nonetheless, we propose that this pipeline is the basis for a standardized approach to the analysis of NGS reads produced from the ASFV by using either combinations of long and short reads, or short reads only. In either case, it helps obtain more accurate ASFV genomes and solves the problem of incompatible annotations in the field. We also hope this will encourage research and diagnostic laboratories to undertake whole genome sequencing and to best utilize available raw sequencing data to further our understanding of the epidemiology, evolution, and genomics of this devastating pathogen.

## Figures and Tables

**Table 1:** Reference database isolates.

GenBank Accession	Isolate
U18466	BA71V
AM712239	Benin
AM712240	OURT_88_3
AY261360	Kenya_1950
AY261361	Malawi_Lil_20_1
AY261362	Mkuzi_1979
AY261363	Pret_96_4
AY261364	Tengani62
AY261365	Warmbaths
AY261366	Warthog
FN557520	E75
KM111294	Ken05
KM111295	Ken06Bus
KM262844	L60
KM262845	NHV
KP055815	BA71
FR682468	Georgia_2007
LR899131	Ken_rie1
LS478113	Estonia2014
MN318203	LIV_5_40
MN394630	SPEC_57
MN630494	Zaire
MN641877	RSA_2_2004
MT956648	Uvira_B53
MW856067	BUR_18_Rutana
MW856068	MAL_19_Karonga
MZ202520	K49
OM249788	KK262
ON400500	YNFN202103
ON409981	TAN_08_Mazimbu
ON409982	TAN_17_Mbagala
OP672342	Nigeria-RV502
OQ504954	Henan/123014/2022

**Table 2:** Host-filtering analysis on different isolates.

Isolate (Biotype)	Reference Genome and Reference	Total Number of Illumina Paired-Reads (% Compared to Post Trim)			Average Coverage	
		Post-Trim	Trimmed, Mapped to Reference, and Extracted	Trimmed, Filtered, Mapped to Reference, and Extracted	Trimmed, Mapped to Reference	Trimmed, Filtered, and Mapped to Reference
ASFV Georgia 2007/1 (2)	FR682468, [1,18]	6,708,139	173,922 (2.59%)	173,886 (2.59%)	158	158
RSA/2/2008 ^a^ (3)	MN336500, [38]	4,575,230	3,256,629 (71.18%)	3,212,202 (70.21%)	3183	3140
Cameroon/2023/SBP30 (1)	PP592890, [39]	1,231,886	50,526 (4.10%)	49796 (4.04%)	91	89
Malawi Lil 80 (6)	AY261361	75,762,615.00	2,631,494 (3.47%)	2,586,133 (3.41%)	2070	2034

^a^ SRA: SRR10282409.

## Data Availability

All data used in this study are from publicly available databases, and the sequencing pipeline described is on GitHub. https://github.com/Global-ASFV-Research-Alliance/ASFV_Pipeline/tree/main (accessed on 5 May 2024).

## Supplementary Materials

The following supporting information can be downloaded at: https://www.mdpi.com/article/10.3390/v16081293/s1.
