# Supplementary material for: A Standardized Pipeline for Assembly and Annotation of African Swine Fever Virus Genome
Source: Viruses. 2024 Aug 13;16(8):1293. doi: 10.3390/v16081293 (PMC11359534; doi:10.3390/v16081293)

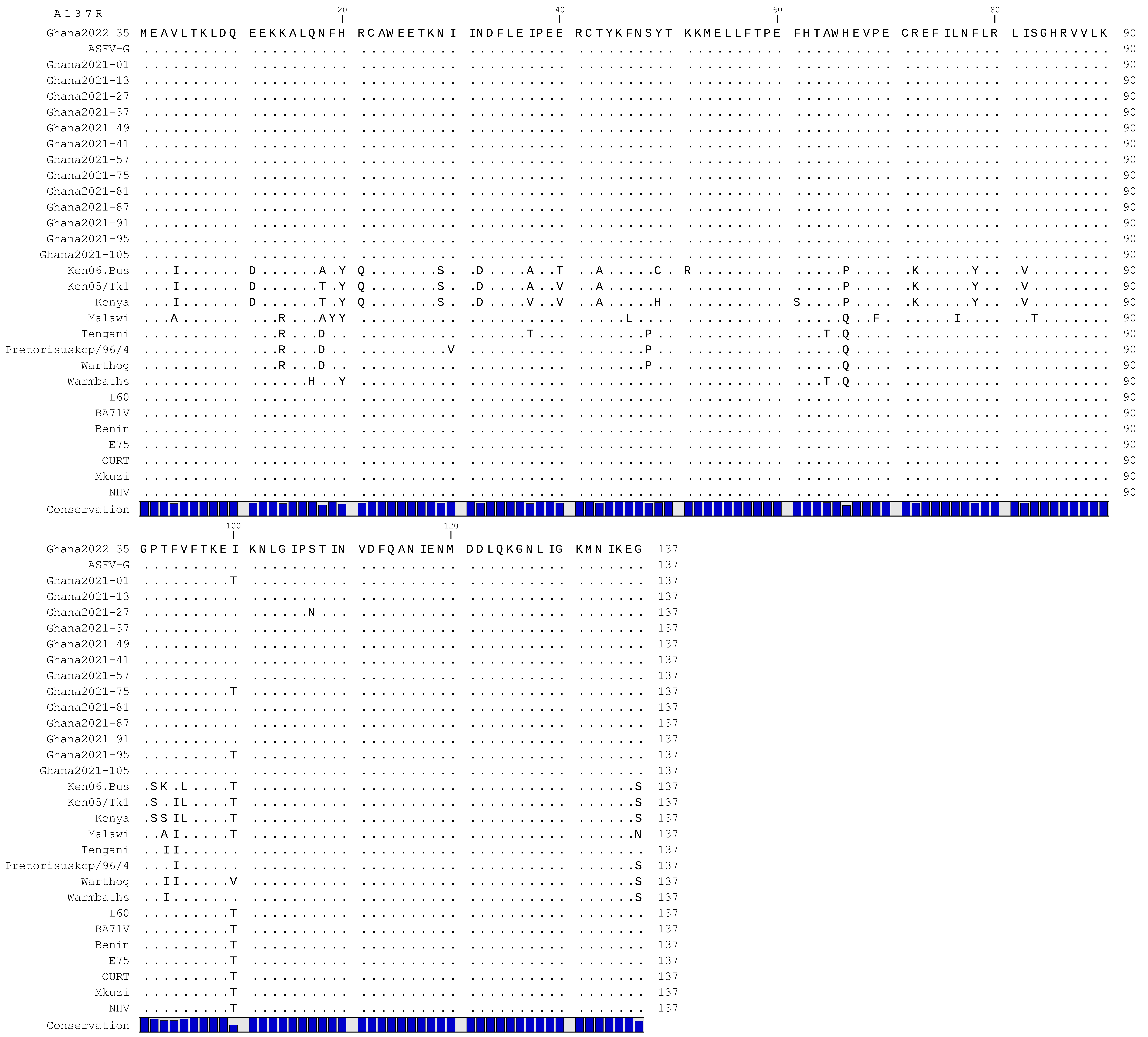

A137R
Ghana2022-35
MEAVLTKLDQ EEKKALQNFH RCAWEETKNI INDFLEIPEE RCTYKFNSYT KKMELLFTPE FHTAWHEVPE CREFILNFLR LISGHRVVLK 90
GPTFVFTKEI KNLGIPSTIN VDFQANIENM DDLQKGNLIG KMNIKEG 137
ASFV-G
Ghana2021-01
Ghana2021-13
Ghana2021-27
Ghana2021-37
Ghana2021-49
Ghana2021-41
Ghana2021-57
Ghana2021-75
Ghana2021-81
Ghana2021-87
Ghana2021-91
Ghana2021-95
Ghana2021-105
Ken06.Bus
Ken05/Tk1
Kenya
Malawi
Tengani
Pretorisuskop/96/4
Warthog
Warmbaths
L60
BA71V
Benin
E75
OURT
Mkuzi
NHV
Conservation

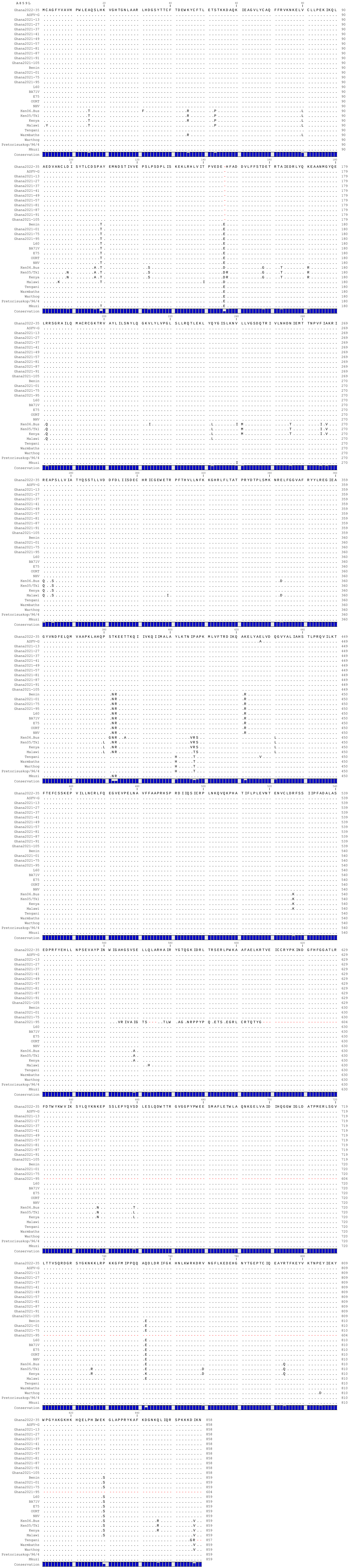
A859L

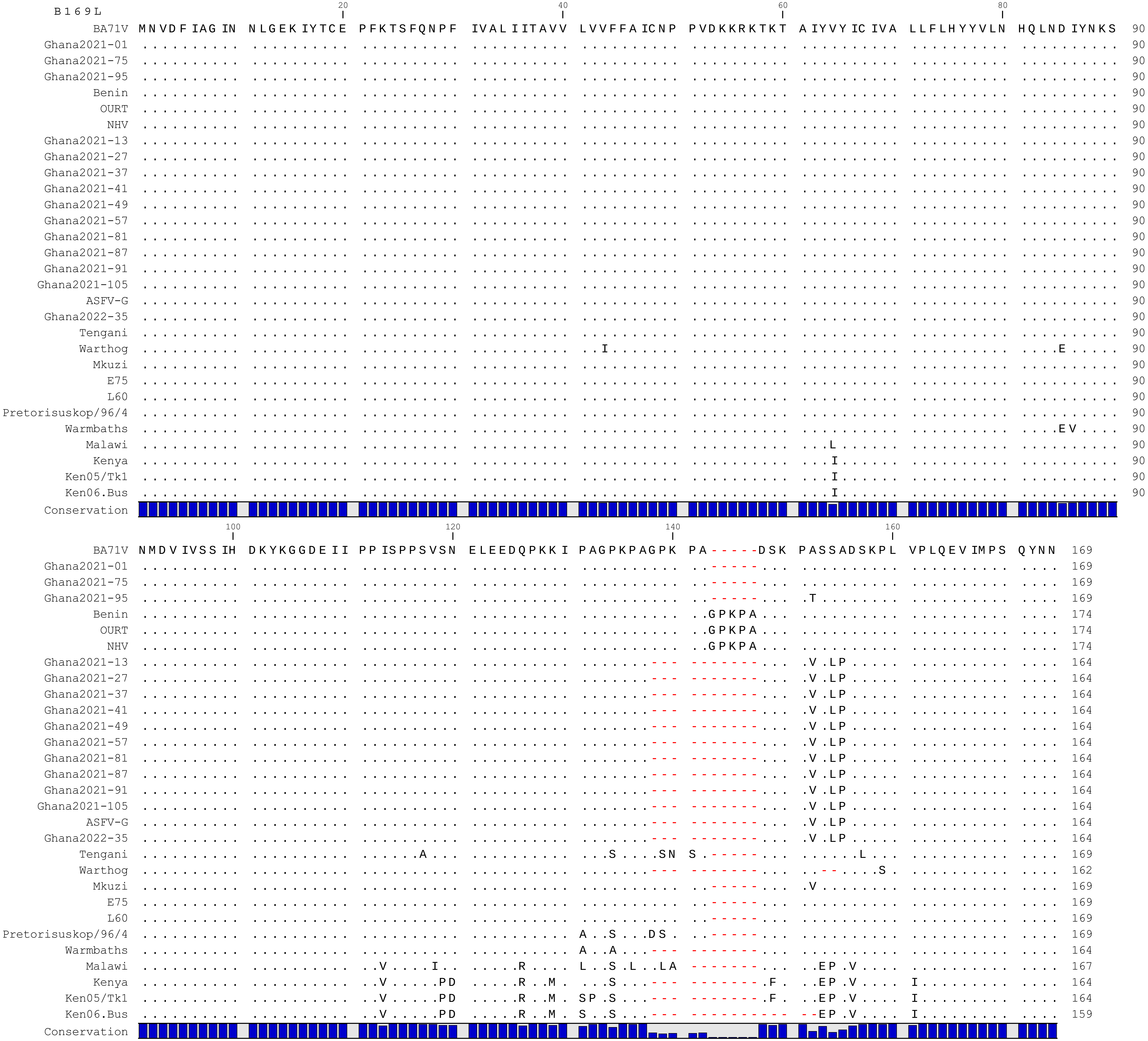

B169L
BA71V MNVDFIAGIN NLGEKIYTCE PFKTSFQNPF IVALIITAVV LVVFFAICNP PVDKKRKTKT AIYVYICIVA LLFLHYYVLN HQLNDIYNKS 90
BA71V NMDVIVSSIH DKYKGGDEII PPISPPSVSN ELEEDQPKKI PAGPKPAGPK PA-----DSK PASSADSKPL VPLQEVIMPS QYNN 169
Ghana2021-01
Ghana2021-75
Ghana2021-95
Benin
OURT
NHV
Ghana2021-13
Ghana2021-27
Ghana2021-37
Ghana2021-41
Ghana2021-49
Ghana2021-57
Ghana2021-81
Ghana2021-87
Ghana2021-91
Ghana2021-105
ASFV-G
Ghana2022-35
Tengani
Warthog
Mkuzi
E75
L60
Pretorisuskop/96/4
Warmbaths
Malawi
Kenya
Ken05/Tk1
Ken06.Bus
Conservation

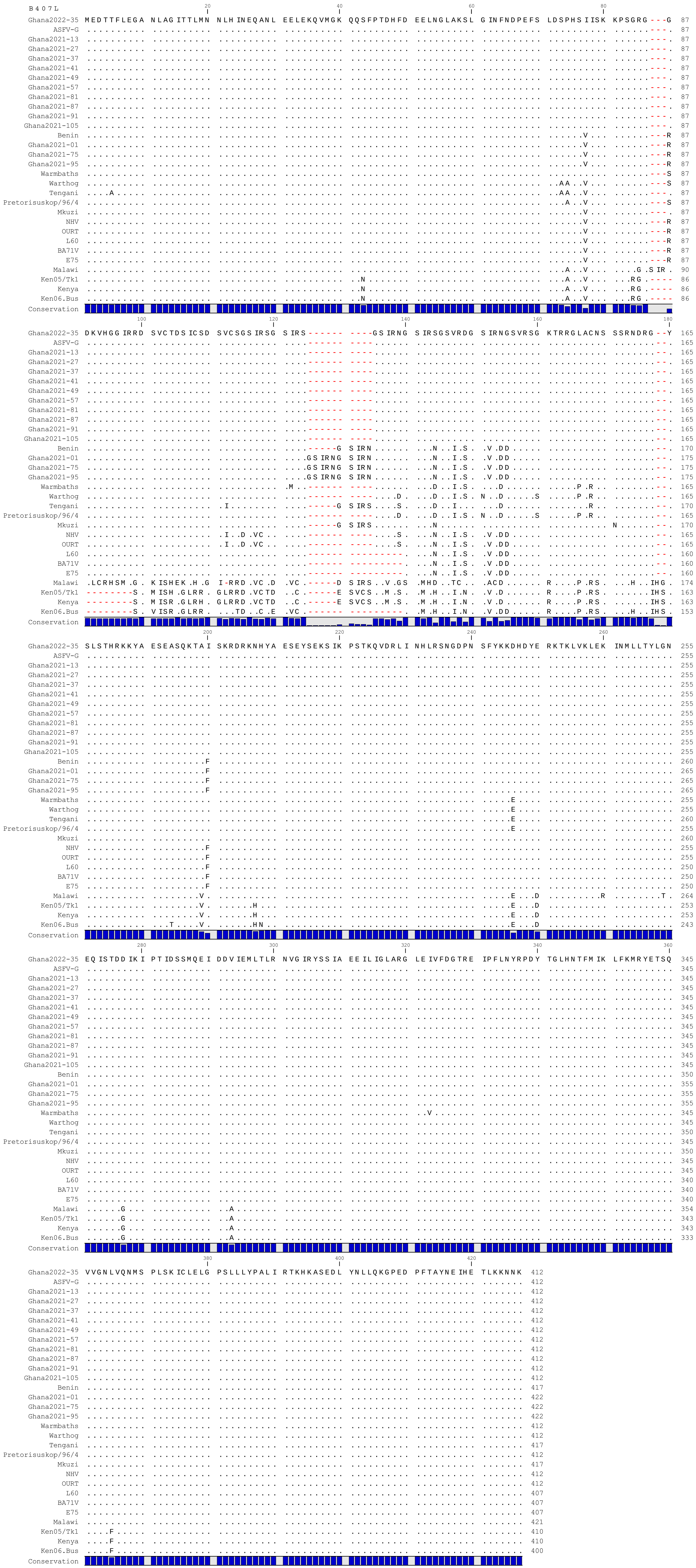
B407L
Ghana2022-35
ASFV-G
Benin
Warmbaths
Warthog
Tengani
Pretorisuskop/96/4
Mkuzi
NHV
OURT
L60
BA71V
E75
Malawi
Ken05/Tk1
Kenya
Ken06.Bus
Conservation

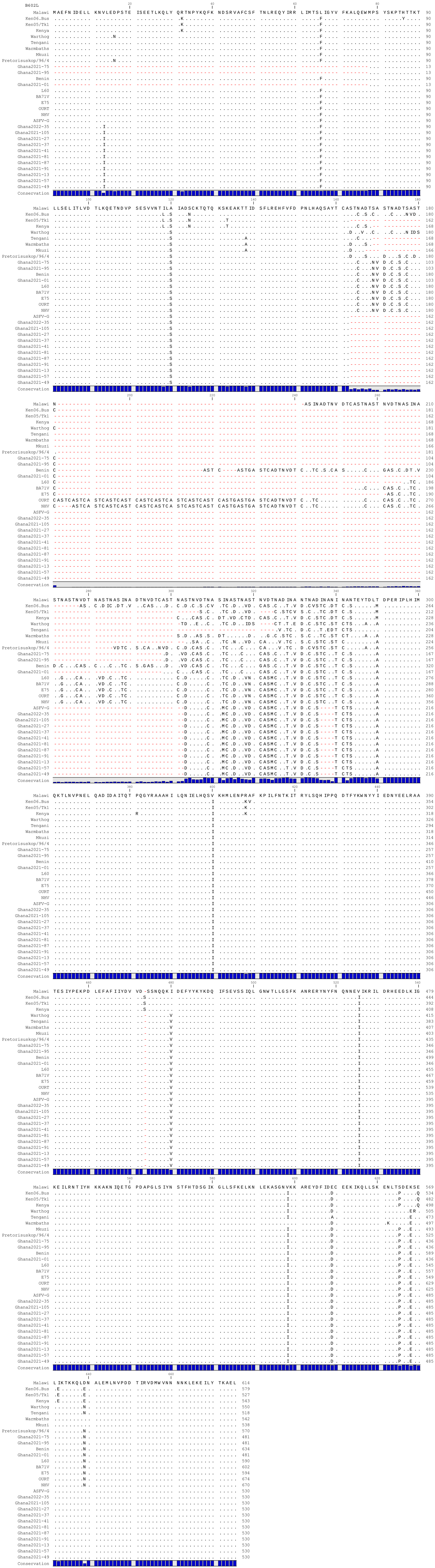

B602L
Conservation

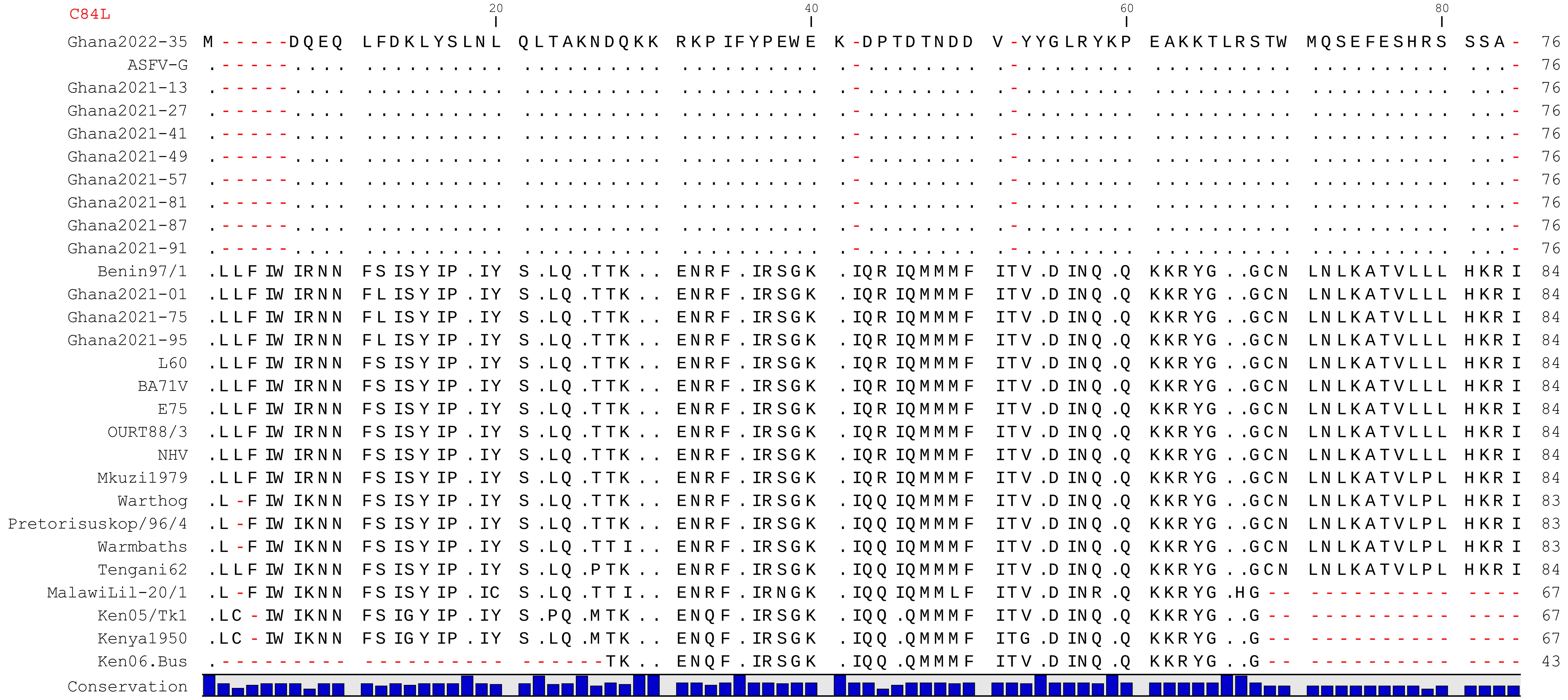

C84L
20
40
60
80
Ghana2022-35 M-----DQEQ LFDKLYSLNL QLTAKNDQKK RKPIFYPEWE K-DPTDTNDD V-YYGLRYKP EAKKTLRSTW MQSEFESHRS SSA- 76
ASFV-G .-----.... ....-........ .-........ ....- 76
Ghana2021-13 .-----.... ....-........ .-........ ....- 76
Ghana2021-27 .-----.... ....-........ .-........ ....- 76
Ghana2021-41 .-----.... ....-........ .-........ ....- 76
Ghana2021-49 .-----.... ....-........ .-........ ....- 76
Ghana2021-57 .-----.... ....-........ .-........ ....- 76
Ghana2021-81 .-----.... ....-........ .-........ ....- 76
Ghana2021-87 .-----.... ....-........ .-........ ....- 76
Ghana2021-91 .-----.... ....-........ .-........ ....- 76
Benin97/1 .LLFIWIRNN FSISYIP.IY S.LQ.TTK.. ENRF.IRSGK .IQRIQMMMF ITV.DINQ.Q KKRYG..GCN LNLKATVLLL HKRI 84
Ghana2021-01 .LLFIWIRNN FLISYIP.IY S.LQ.TTK.. ENRF.IRSGK .IQRIQMMMF ITV.DINQ.Q KKRYG..GCN LNLKATVLLL HKRI 84
Ghana2021-75 .LLFIWIRNN FLISYIP.IY S.LQ.TTK.. ENRF.IRSGK .IQRIQMMMF ITV.DINQ.Q KKRYG..GCN LNLKATVLLL HKRI 84
Ghana2021-95 .LLFIWIRNN FLISYIP.IY S.LQ.TTK.. ENRF.IRSGK .IQRIQMMMF ITV.DINQ.Q KKRYG..GCN LNLKATVLLL HKRI 84
L60 .LLFIWIRNN FSISYIP.IY S.LQ.TTK.. ENRF.IRSGK .IQRIQMMMF ITV.DINQ.Q KKRYG..GCN LNLKATVLLL HKRI 84
BA71V .LLFIWIRNN FSISYIP.IY S.LQ.TTK.. ENRF.IRSGK .IQRIQMMMF ITV.DINQ.Q KKRYG..GCN LNLKATVLLL HKRI 84
E75 .LLFIWIRNN FSISYIP.IY S.LQ.TTK.. ENRF.IRSGK .IQRIQMMMF ITV.DINQ.Q KKRYG..GCN LNLKATVLLL HKRI 84
OURT88/3 .LLFIWIRNN FSISYIP.IY S.LQ.TTK.. ENRF.IRSGK .IQRIQMMMF ITV.DINQ.Q KKRYG..GCN LNLKATVLLL HKRI 84
NHV .LLFIWIRNN FSISYIP.IY S.LQ.TTK.. ENRF.IRSGK .IQRIQMMMF ITV.DINQ.Q KKRYG..GCN LNLKATVLLL HKRI 84
Mkuzi1979 .LLFIWIRNN FSISYIP.IY S.LQ.TTK.. ENRF.IRSGK .IQRIQMMMF ITV.DINQ.Q KKRYG..GCN LNLKATVLPL HKRI 84
Warthog .L-FIWIKNN FSISYIP.IY S.LQ.TTK.. ENRF.IRSGK .IQQIQMMMF ITV.DINQ.Q KKRYG..GCN LNLKATVLPL HKRI 83
Pretorisuskop/96/4 .L-FIWIKNN FSISYIP.IY S.LQ.TTK.. ENRF.IRSGK .IQQIQMMMF ITV.DINQ.Q KKRYG..GCN LNLKATVLPL HKRI 83
Warmbaths .L-FIWIKNN FSISYIP.IY S.LQ.TTI.. ENRF.IRSGK .IQQIQMMMF ITV.DINQ.Q KKRYG..GCN LNLKATVLPL HKRI 83
Tengani62 .LLFIWIKNN FSISYIP.IY S.LQ.PTK.. ENRF.IRSGK .IQQIQMMMF ITV.DINQ.Q KKRYG..GCN LNLKATVLPL HKRI 84
MalawiLil-20/1 .L-FIWIKNN FSISYIP.IC S.LQ.TTI.. ENRF.IRNGK .IQQIQMMLF ITV.DINR.Q KKRYG.HG-- ---------- ---- 67
Ken05/Tk1 .LC-IWIKNN FSIGYIP.IY S.PQ.MTK.. ENQF.IRSGK .IQQ.QMMMF ITV.DINQ.Q KKRYG..G-- ---------- ---- 67
Kenya1950 .LC-IWIKNN FSIGYIP.IY S.LQ.MTK.. ENQF.IRSGK .IQQ.QMMMF ITG.DINQ.Q KKRYG..G-- ---------- ---- 67
Ken06.Bus .--------- ---------- ------TK.. ENQF.IRSGK .IQQ.QMMMF ITV.DINQ.Q KKRYG..G-- ---------- ---- 43
Conservation

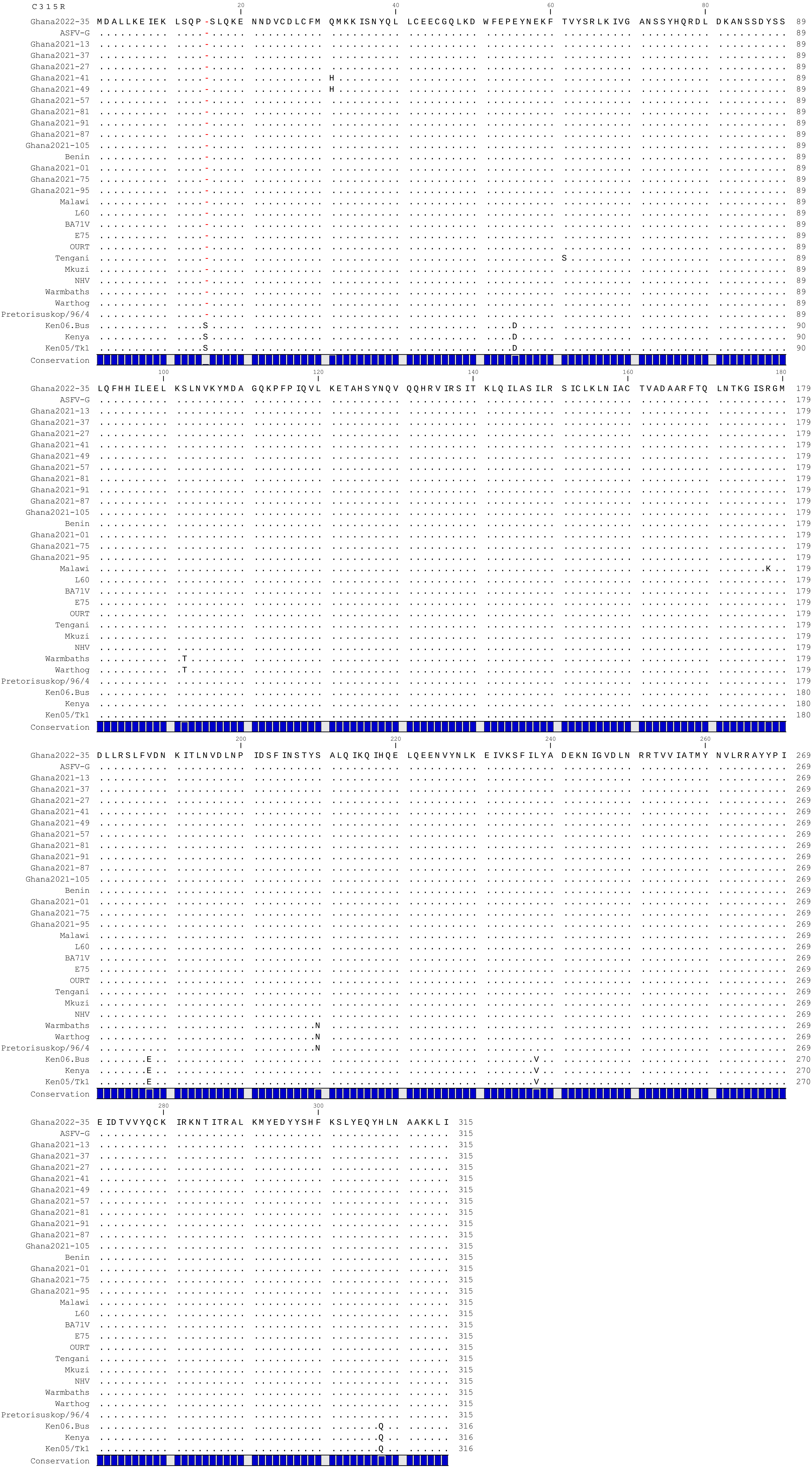
C315R
Ghana2022-35 MDALLKEIEK LSQP-SLQKE NNDVCDLCFM QMKKISNYQL LCEECGQLKD WFEPEYNEKF TVYSRLKIVG ANSSYHQRDL DKANSSDYSS 89
Ghana2022-35 LQFHHILEEL KSLNVKYMDA GQKPFPIQVL KETAHSYNQV QQHRVIRSIT KLQILASILR SICLKLNIAC TVADAARFTQ LNTKGISRGM 179
Ghana2022-35 DLLRSLFVDN KITLNVDLNP IDSFINSTYS ALQIKQIHQE LQEENVYNLK EIVKSFILYA DEKNIGVDLN RRTVVIATMY NVLRRAYYPI 269
Ghana2022-35 EIDTVVYQCK IRKNTITRAL KMYEDYYSHF KSLYEQYHLN AAKKLI 315
ASFV-G
Ghana2021-13
Ghana2021-37
Ghana2021-27
Ghana2021-41
Ghana2021-49
Ghana2021-57
Ghana2021-81
Ghana2021-91
Ghana2021-87
Ghana2021-105
Benin
Ghana2021-01
Ghana2021-75
Ghana2021-95
Malawi
L60
BA71V
E75
OURT
Tengani
Mkuzi
NHV
Warmbaths
Warthog
Pretorisuskop/96/4
Ken06.Bus
Kenya
Ken05/Tk1
Conservation

C475L

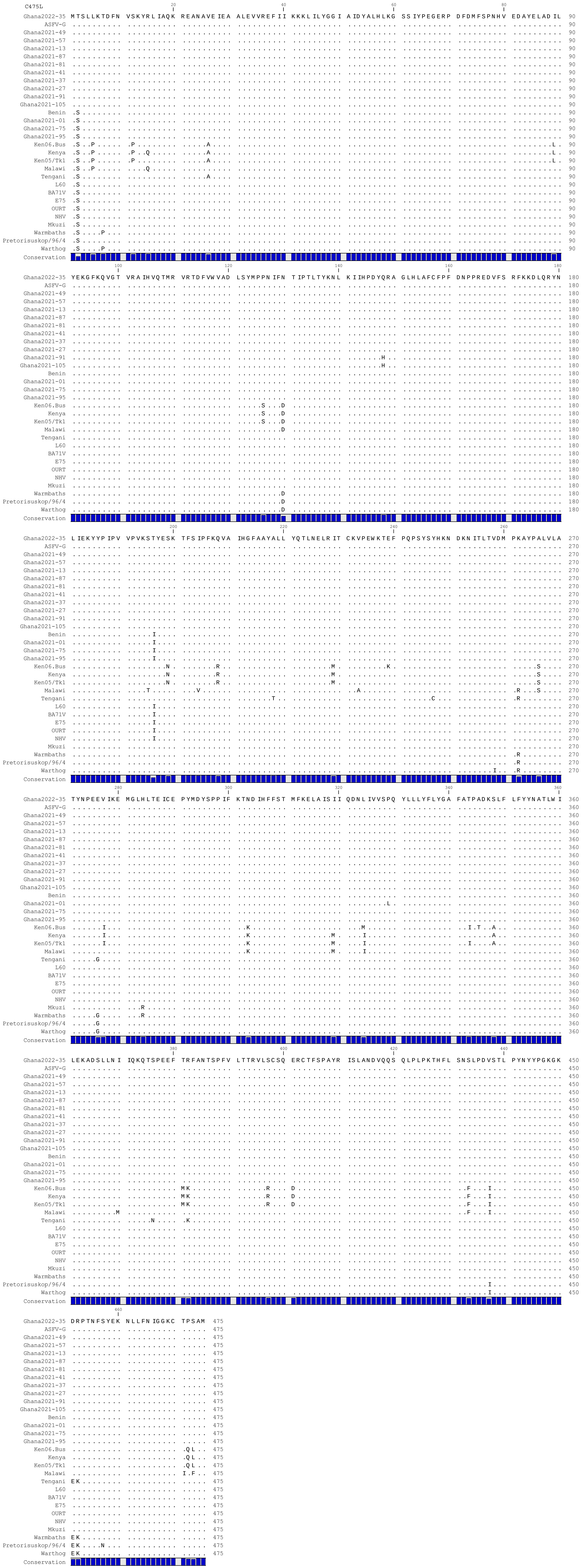

CT178

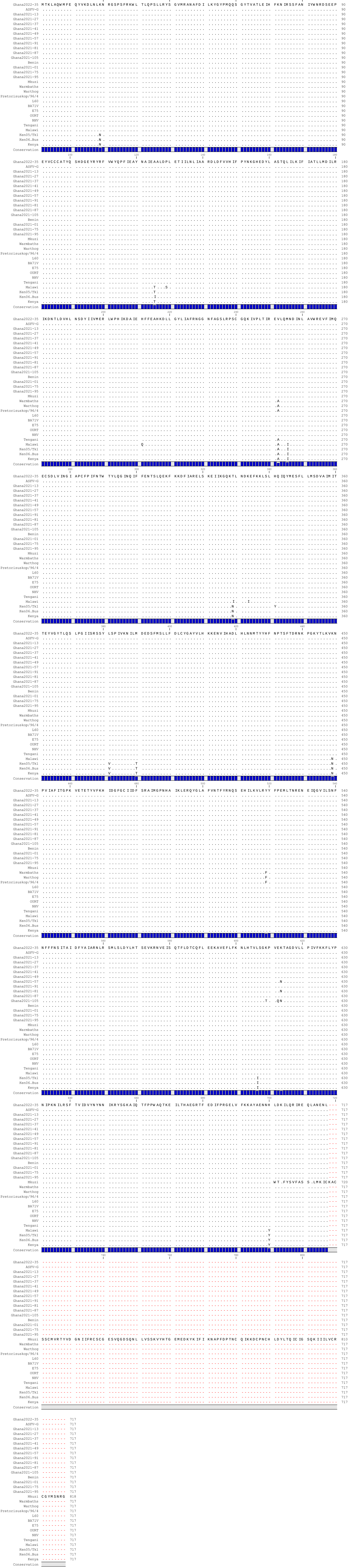

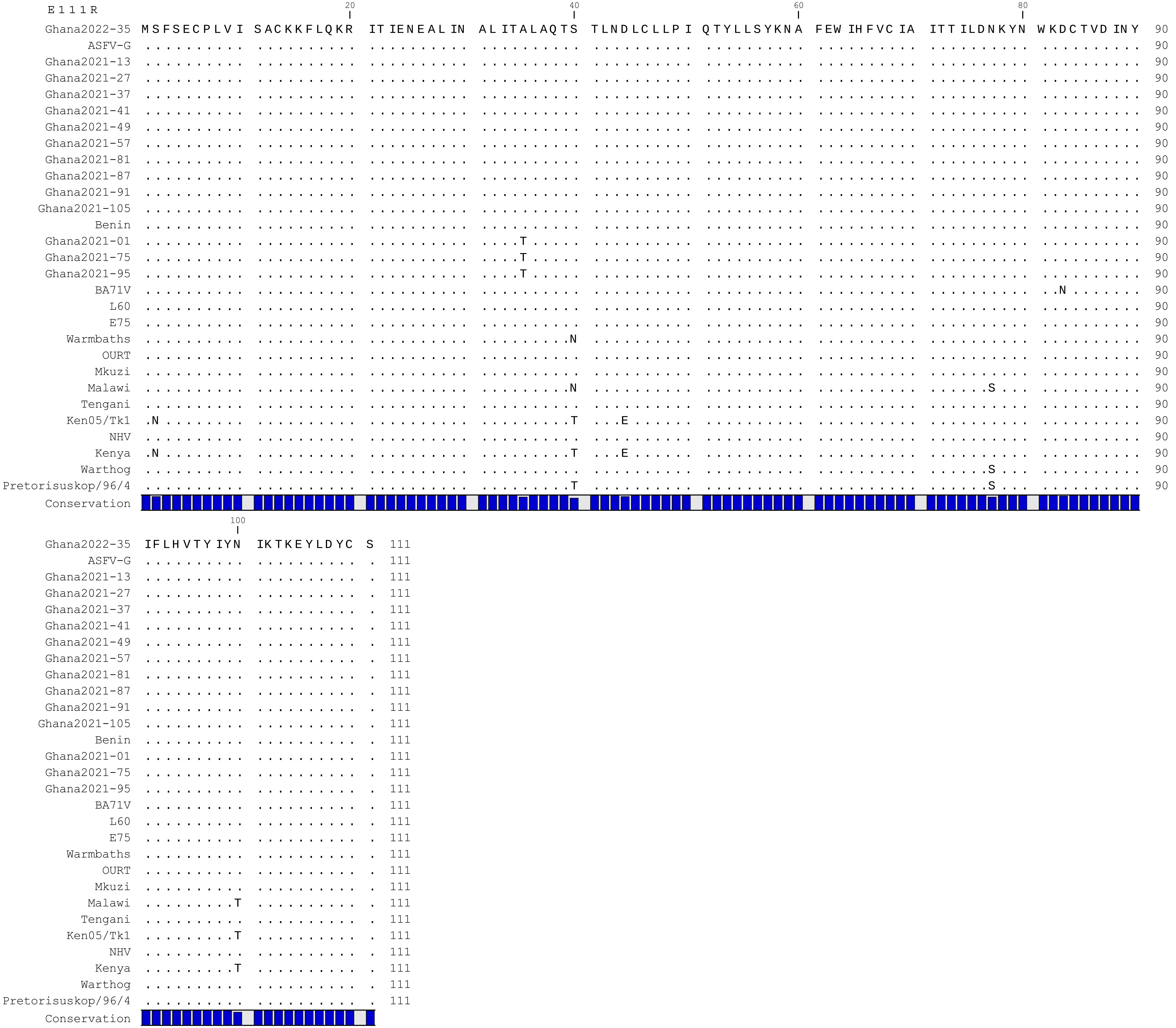
E111R
20
40
60
80
Ghana2022-35 MSFSECPLVI SACKKFLQKR ITIENEALIN ALITALAQTS TLNDLCLLPI QTYLLSYKNA FEWIHFVCIA ITTILDNKYN WKDCTVDINY 90
ASFV-G 90
Ghana2021-13 90
Ghana2021-27 90
Ghana2021-37 90
Ghana2021-41 90
Ghana2021-49 90
Ghana2021-57 90
Ghana2021-81 90
Ghana2021-87 90
Ghana2021-91 90
Ghana2021-105 90
Benin 90
Ghana2021-01 ....T..... 90
Ghana2021-75 ....T..... 90
Ghana2021-95 ....T..... 90
BA71V ....N....... 90
L60 90
E75 90
Warmbaths ....N 90
OURT 90
Mkuzi 90
Malawi ....N ....S... 90
Tengani 90
Ken05/Tk1 .N........ ....T ...E...... 90
NHV 90
Kenya .N........ ....T ...E...... 90
Warthog ....S... 90
Pretorisuskop/96/4 ....T ....S... 90
Conservation
100
Ghana2022-35 IFLHVTYIYN IKTKEYLDYC S 111
ASFV-G 111
Ghana2021-13 111
Ghana2021-27 111
Ghana2021-37 111
Ghana2021-41 111
Ghana2021-49 111
Ghana2021-57 111
Ghana2021-81 111
Ghana2021-87 111
Ghana2021-91 111
Ghana2021-105 111
Benin 111
Ghana2021-01 111
Ghana2021-75 111
Ghana2021-95 111
BA71V 111
L60 111
E75 111
Warmbaths 111
OURT 111
Mkuzi 111
Malawi ....T 111
Tengani 111
Ken05/Tk1 ....T 111
NHV 111
Kenya ....T 111
Warthog 111
Pretorisuskop/96/4 111
Conservation

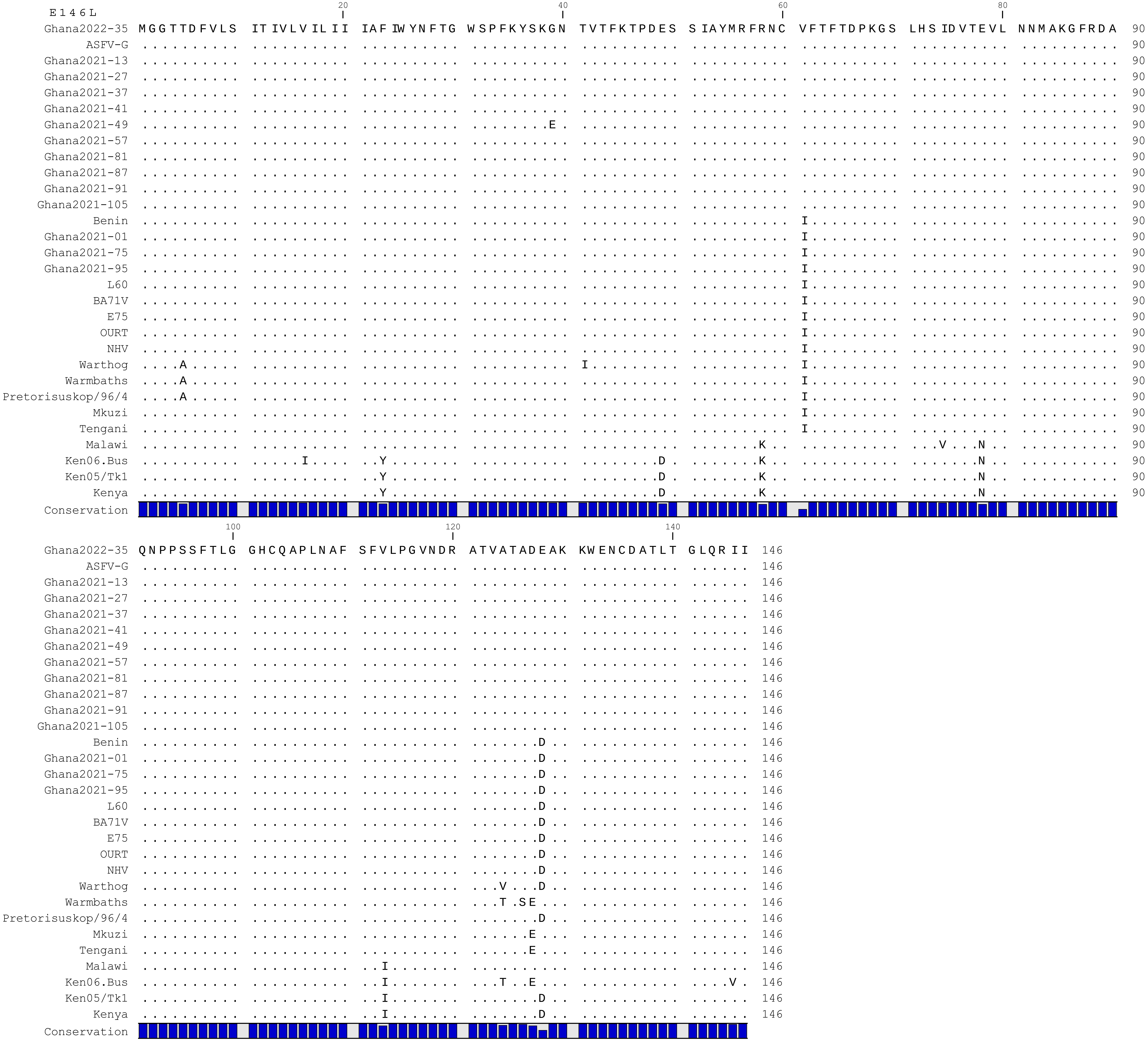
E146L
Ghana2022-35 MGGTTDFVLS ITIVLVILII IAFIWYNFTG WSPFKYSKGN TVTFKTPDES SIAYMRFRNC VFTFTDPKGS LHSIDVTEVL NNMAKGFRDA 90
Ghana2022-35 QNPPSSFTLG GHCQAPLNAF SFVLPGVNDR ATVATADEAK KWENCDATLT GLQRII 146
ASFV-G
Ghana2021-13
Ghana2021-27
Ghana2021-37
Ghana2021-41
Ghana2021-49
Ghana2021-57
Ghana2021-81
Ghana2021-87
Ghana2021-91
Ghana2021-105
Benin
Ghana2021-01
Ghana2021-75
Ghana2021-95
L60
BA71V
E75
OURT
NHV
Warthog
Warmbaths
Pretorisuskop/96/4
Mkuzi
Tengani
Malawi
Ken06.Bus
Ken05/Tk1
Kenya
Conservation

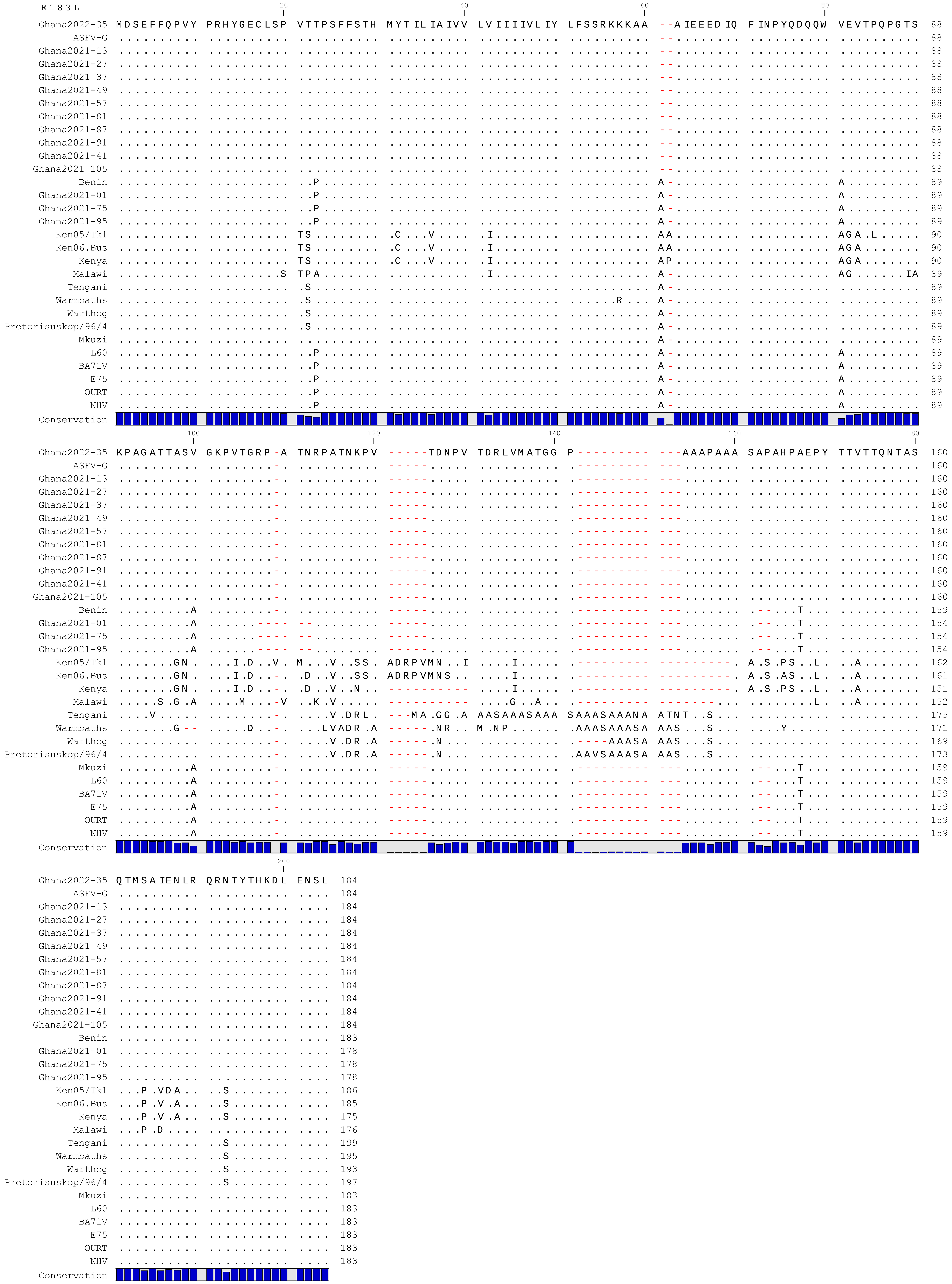
E183L
Ghana2022-35 MDSEFFQPVY PRHYGECLSP VTTPSFFSTH MYTILIAIVV LVIIIIVLIY LFSSRKKKAA --AIEEEDIQ FINPYQDQQW VEVTPQPGTS 88
Ghana2022-35 KPAGATTASV GKPVTGRP-A TNRPATNKPV -----TDNPV TDRLVMATGG P---------- ---AAAPAAA SAPAHPAEPY TTVTTQNTAS 160
Ghana2022-35 QTMSAIENLR QRNTYTHKDL ENSL 184
ASFV-G
Ghana2021-13
Ghana2021-27
Ghana2021-37
Ghana2021-49
Ghana2021-57
Ghana2021-81
Ghana2021-87
Ghana2021-91
Ghana2021-41
Ghana2021-105
Benin
Ghana2021-01
Ghana2021-75
Ghana2021-95
Ken05/Tk1
Ken06.Bus
Kenya
Malawi
Tengani
Warmbaths
Warthog
Pretorisuskop/96/4
Mkuzi
L60
BA71V
E75
OURT
NHV
Conservation

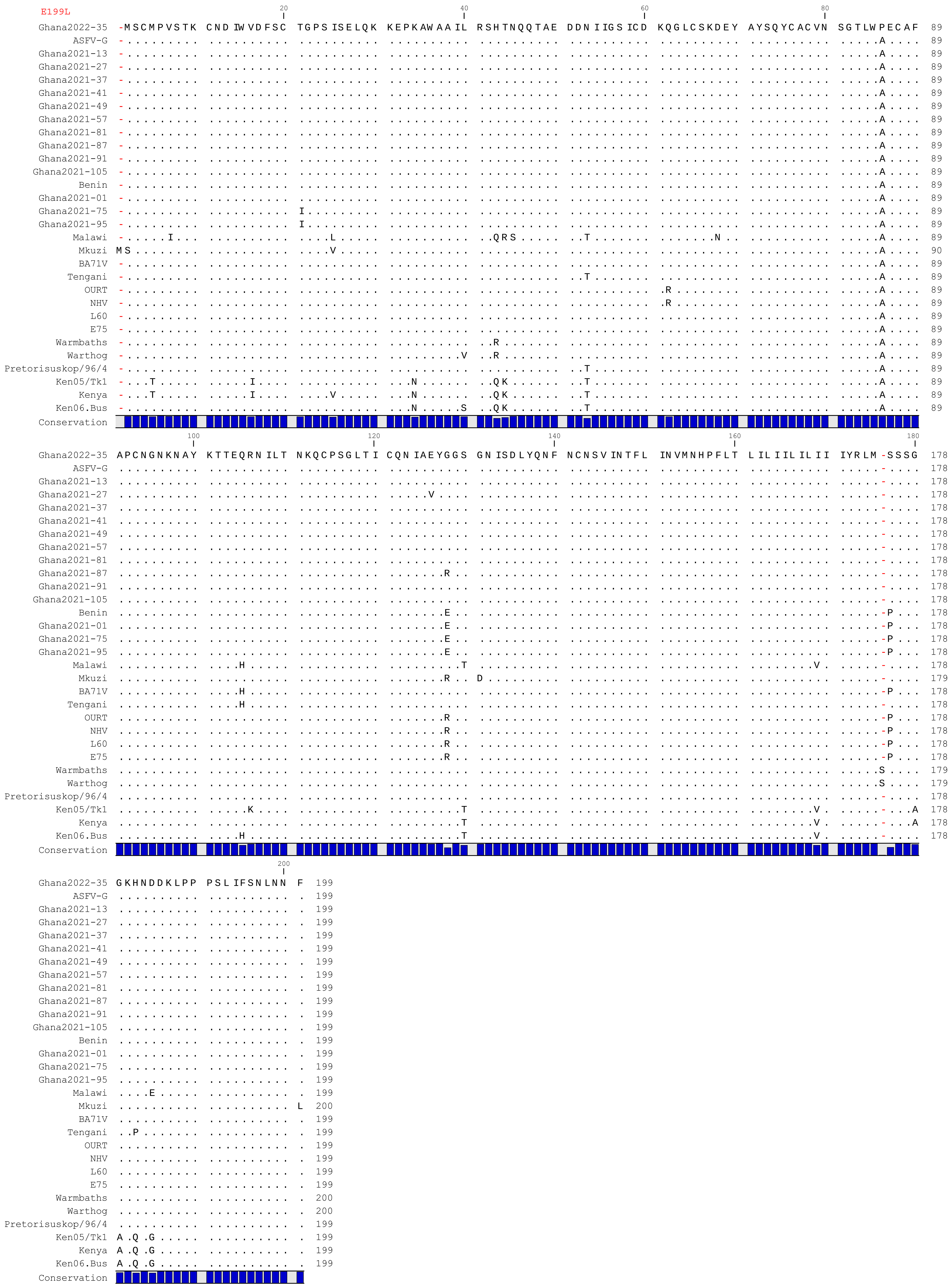
E199L
Ghana2022-35 -MSCMPVSTK CNDIWVDFSC TGPSISELQK KEPKAWAAIL RSHTNQQTAE DDNIIGSICD KQGLCSKDEY AYSQYCACVN SGTLWPECAF 89
Ghana2022-35 APCNGNKNAY KTTEQRNILT NKQCPSGLTI CQNIAEYGGS GNISDLYQNF NCNSVINTFL INVMNHPFLT LILIILILII IYRLM-SSSG 178
Ghana2022-35 GKHNDDKLPP PSLIFSNLNN F 199
ASFV-G
Ghana2021-13
Ghana2021-27
Ghana2021-37
Ghana2021-41
Ghana2021-49
Ghana2021-57
Ghana2021-81
Ghana2021-87
Ghana2021-91
Ghana2021-105
Benin
Ghana2021-01
Ghana2021-75
Ghana2021-95
Malawi
Mkuzi
BA71V
Tengani
OURT
NHV
L60
E75
Warmbaths
Warthog
Pretorisuskop/96/4
Ken05/Tk1
Kenya
Ken06.Bus
Conservation

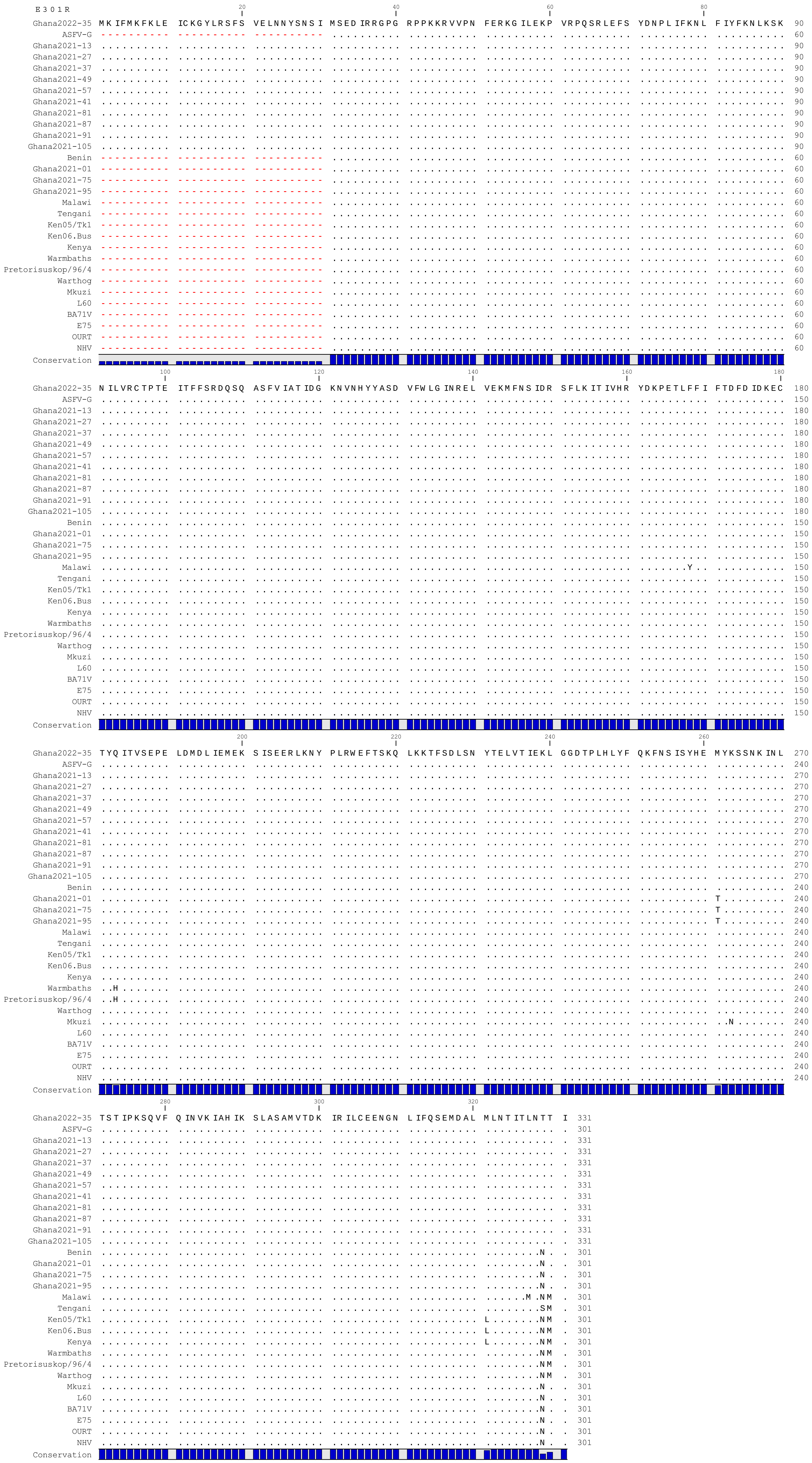
E301R
Ghana2022-35 MKIFMKFKLE ICKGYLRSFS VELNNYSNSI MSEDIRRGPG RPPKKRVVPN FERKGILEKP VRPQSRLEFS YDNPLIFKNL FIYFKNLKSK 90
Ghana2022-35 NILVRCTPTE ITFFSRDQSQ ASFVIATIDG KNVNHYYASD VFWLGINREL VEKMFNSIDR SFLKITIVHR YDKPETLFFI FTDFDIDKEC 180
Ghana2022-35 TYQITVSEPE LDMDLIEMEK SISEERLKNY PLRWEFTSKQ LKKTFSDLSN YTELVTIEKL GGDTPLHLYF QKFNSISYHE MYKSSNKINL 270
Ghana2022-35 TSTIPKSQVF QINVKIAHIK SLASAMVTDK IRILCEENGN LIFQSEMDAL MLNTITLNTT I 331
Conservation

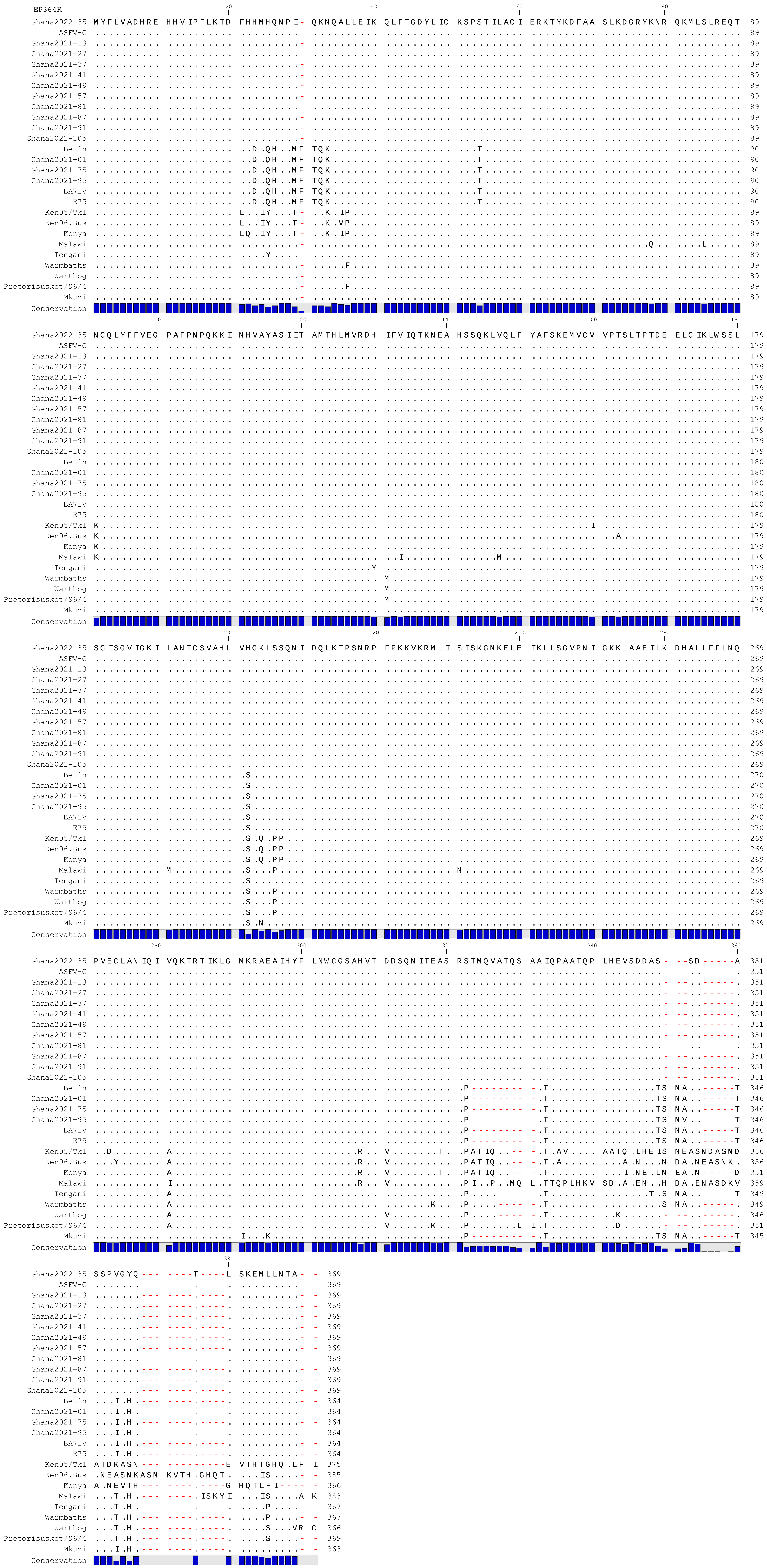
EP364R
Ghana2022-35
ASFV-G
Ghana2021-13
Ghana2021-27
Ghana2021-37
Ghana2021-41
Ghana2021-49
Ghana2021-57
Ghana2021-81
Ghana2021-87
Ghana2021-91
Ghana2021-105
Benin
Ghana2021-01
Ghana2021-75
Ghana2021-95
BA71V
E75
Ken05/Tk1
Ken06.Bus
Kenya
Malawi
Tengani
Warmbaths
Warthog
Pretorisuskop/96/4
Mkuzi
Conservation
MYFLVADHRE HHVIPFLKTD FHHMHQNPI- QKNQALLEIK QLFTGDYLIC KSPSTILACI ERKTYKDFAA SLKDGRYKNR QKMLSLREQT 89
NCQLYFFVEG PAFPNPQKKI NHVAYASIIT AMTHLMVRDH IFVIQTKNEA HSSQKLVQLF YAFSKEMVCV VPTSLTPTDE ELCIKLWSSL 179
SGISGVIGKI LANTCSVAHL VHGKLSSQNI DQLKTPSNRP FPKKVKRMLI SISKGNKELE IKLLSGVPNI GKKLAAEILK DHALLFFLNQ 269
PVECLANIQI VQKTRTIKLG MKRAEAIHYF LNWCGSAHVT DDSQNITEAS RSTMQVATQS AAIQPAATQP LHEVSDDAS- --SD-----A 351
SSPVGYQ--- ----T----L SKEMLLNTA- - 369

F1055L

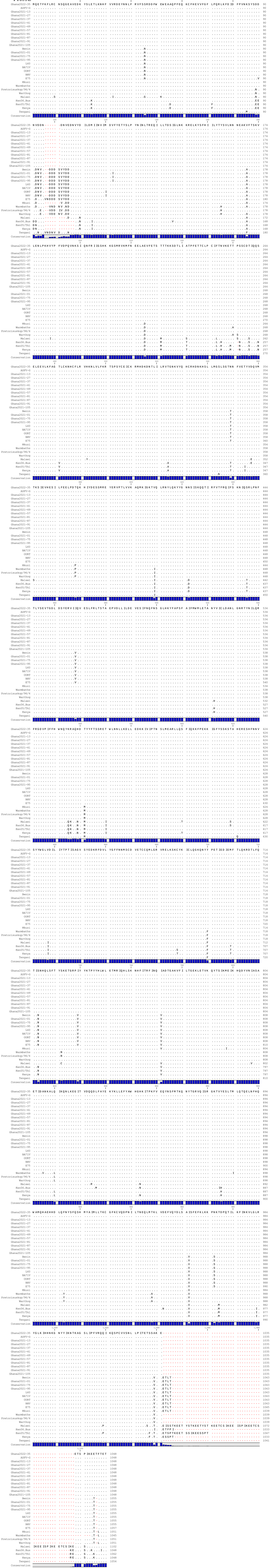

G140L

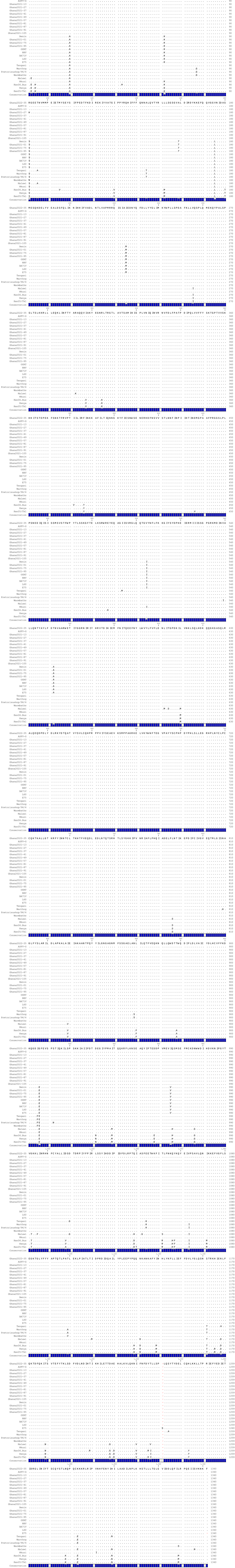

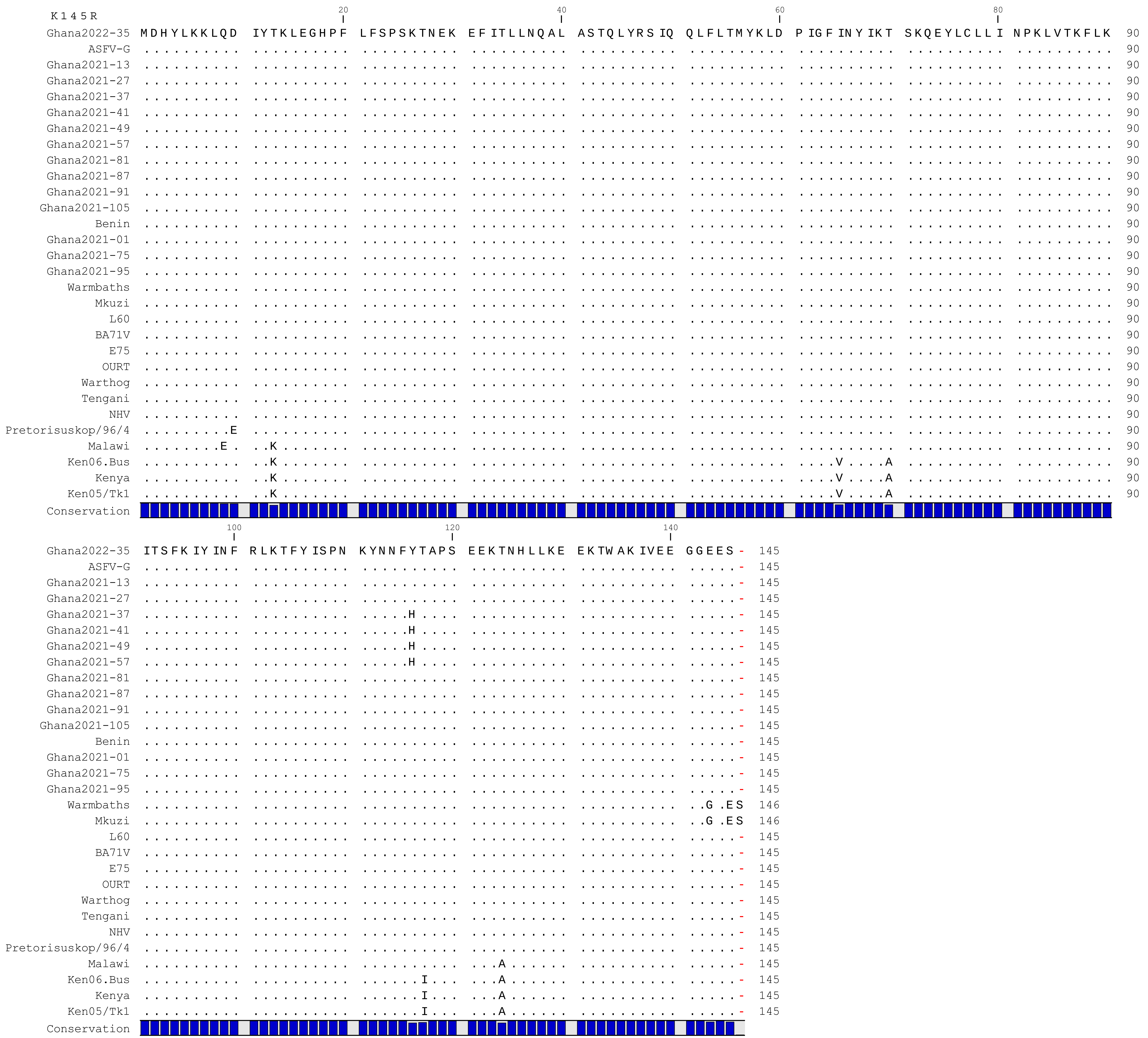
K145R
20 40 60 80
Ghana2022-35 MDHYLKKLQD IYTKLEGHPF LFSPSKTNEK EFITLLNQAL ASTQLYRSIQ QLFLTMYKLD PIGFINYIKT SKQEYLCLLI NPKLVTKFLK 90
ASFV-G 90
Ghana2021-13 90
Ghana2021-27 90
Ghana2021-37 90
Ghana2021-41 90
Ghana2021-49 90
Ghana2021-57 90
Ghana2021-81 90
Ghana2021-87 90
Ghana2021-91 90
Ghana2021-105 90
Benin 90
Ghana2021-01 90
Ghana2021-75 90
Ghana2021-95 90
Warmbaths 90
Mkuzi 90
L60 90
BA71V 90
E75 90
OURT 90
Warthog 90
Tengani 90
NHV 90
Pretorisuskop/96/4 ....E 90
Malawi ....E. ..K....... 90
Ken06.Bus ....K....... ....V....A 90
Kenya ....K....... ....V....A 90
Ken05/Tk1 ....K....... ....V....A 90
Conservation
100 120 140
Ghana2022-35 ITSFKIYINF RLKTFYISPN KYNNFYTAPS EEKTNHLLKE EKTWAKIVEE GGEES- 145
ASFV-G ....- 145
Ghana2021-13 ....- 145
Ghana2021-27 ....- 145
Ghana2021-37 ....H.... ....- 145
Ghana2021-41 ....H.... ....- 145
Ghana2021-49 ....H.... ....- 145
Ghana2021-57 ....H.... ....- 145
Ghana2021-81 ....- 145
Ghana2021-87 ....- 145
Ghana2021-91 ....- 145
Ghana2021-105 ....- 145
Benin ....- 145
Ghana2021-01 ....- 145
Ghana2021-75 ....- 145
Ghana2021-95 ....- 145
Warmbaths ....G.ES 146
Mkuzi ....G.ES 146
L60 ....- 145
BA71V ....- 145
E75 ....- 145
OURT ....- 145
Warthog ....- 145
Tengani ....- 145
NHV ....- 145
Pretorisuskop/96/4 ....- 145
Malawi ....A...... ....- 145
Ken06.Bus ....I... ...A...... ....- 145
Kenya ....I... ...A...... ....- 145
Ken05/Tk1 ....I... ...A...... ....- 145
Conservation

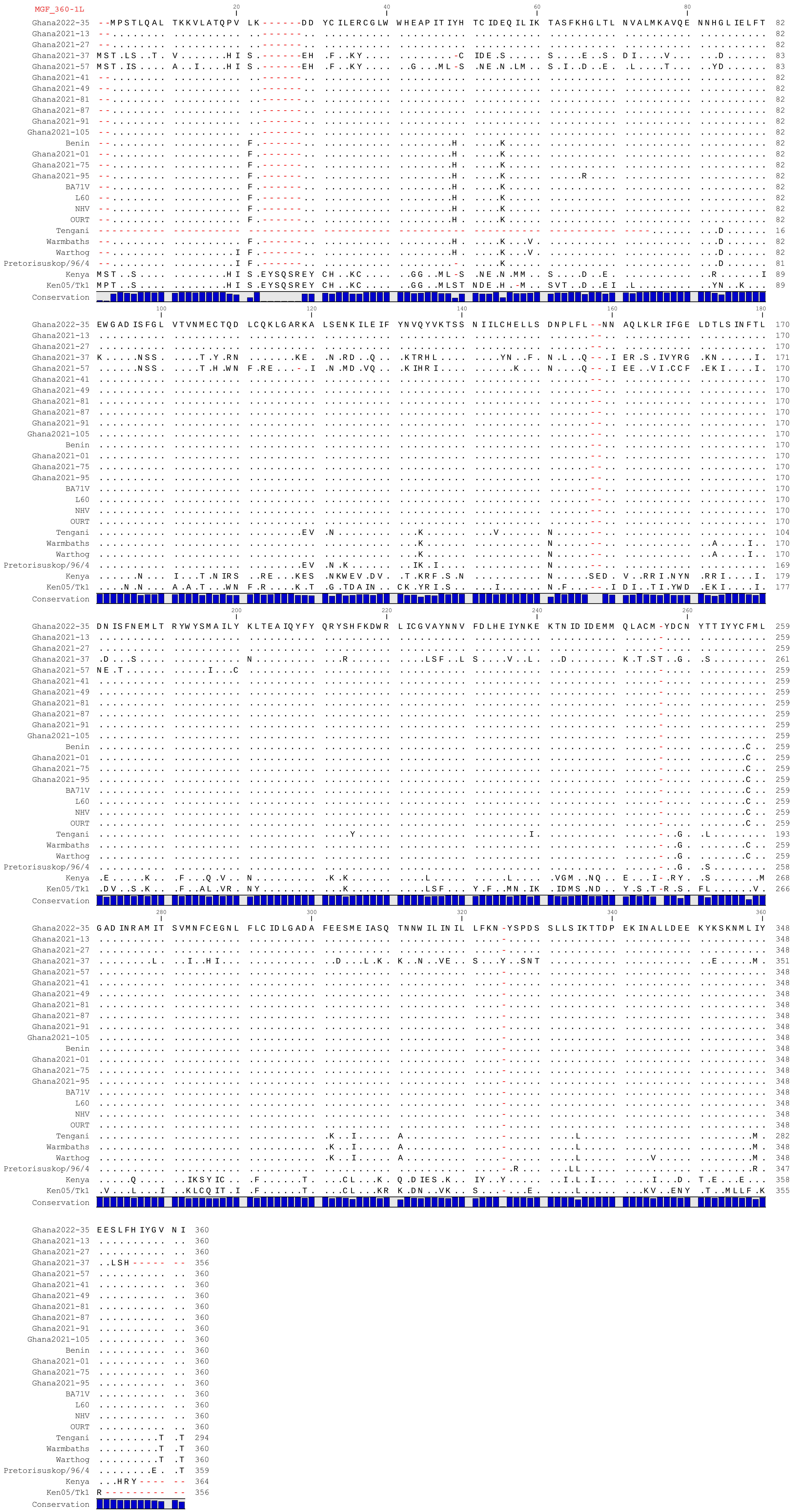
MGF_360-1L
Ghana2022-35
Ghana2021-13
Ghana2021-27
Ghana2021-37
Ghana2021-57
Ghana2021-41
Ghana2021-49
Ghana2021-81
Ghana2021-87
Ghana2021-91
Ghana2021-105
Benin
Ghana2021-01
Ghana2021-75
Ghana2021-95
BA71V
L60
NHV
OURT
Tengani
Warmbaths
Warthog
Pretorisuskop/96/4
Kenya
Ken05/Tk1
Conservation

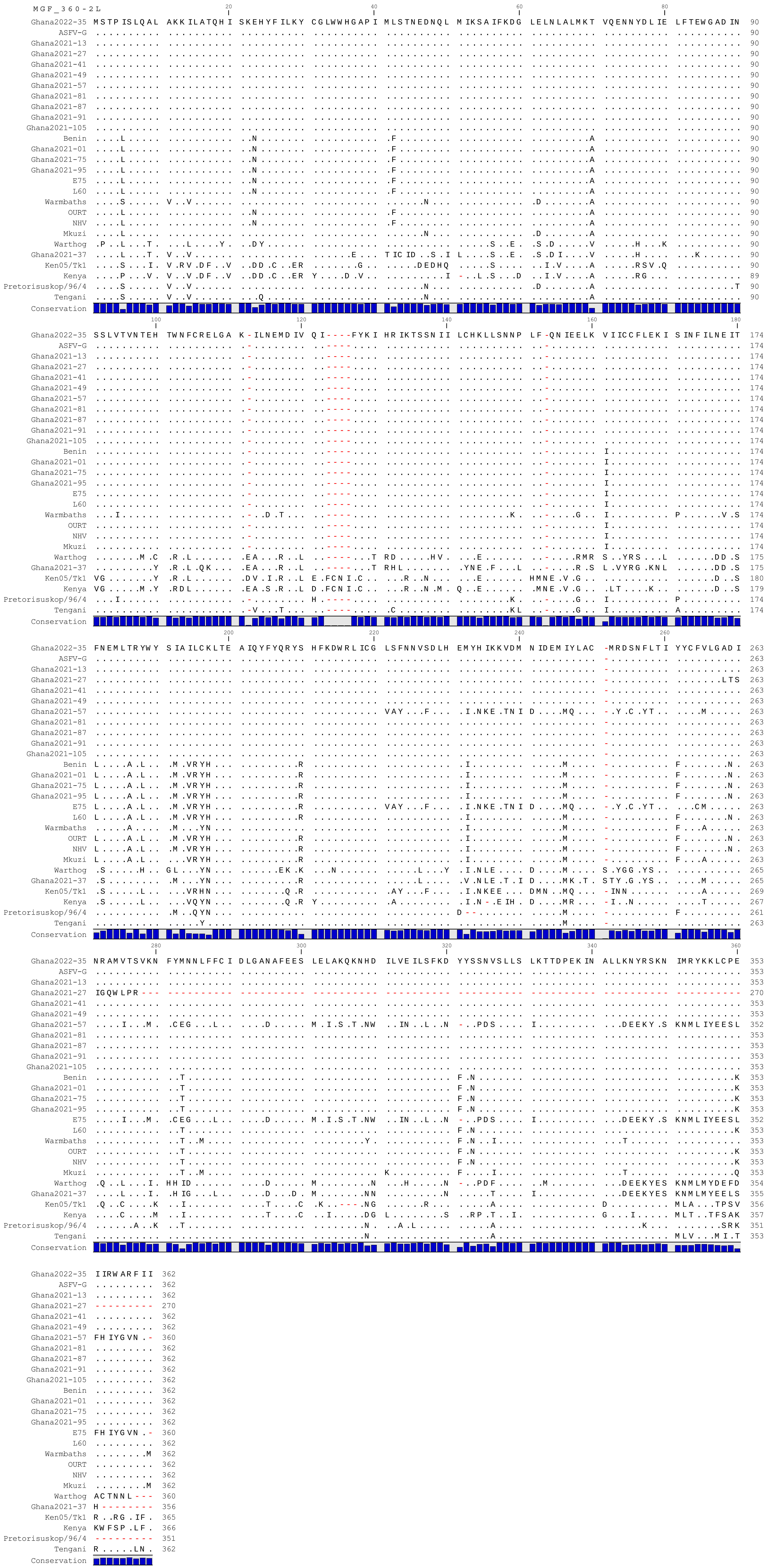

MGF_360-2L
Ghana2022-35 MSTPISLQAL AKKILATQHI SKEHYFILKY CGLWWHGAPI MLSTNEDNQL MIKSAIFKDG LELNLALMKT VQENNYDLIE LFTEWGADIN 90
Ghana2022-35 SSLVTVNTEH TWNFCRELGA K-ILNEMDIV QI----FYKI HRIKTSSNII LCHKLLSNNP LF-QNIEELK VIICCFLEKI SINFILNEIT 174
Ghana2022-35 FNEMLTRYWY SIAILCKLTE AIQYFYQRYS HFKDWRLICG LSFNNVSDLH EMYHIKKVDM NIDEMIYLAC -MRDSNFLTI YYCFVLGADI 263
Ghana2022-35 NRAMVTSVKN FYMNNLFFCI DLGANAFEES LELAKQKNHD ILVEILSFKD YYSSNVSLLS LKTTDPEKIN ALLKNYRSKN IMRYKKLCPE 353
Ghana2022-35 IIRWARFII 362
Conservation

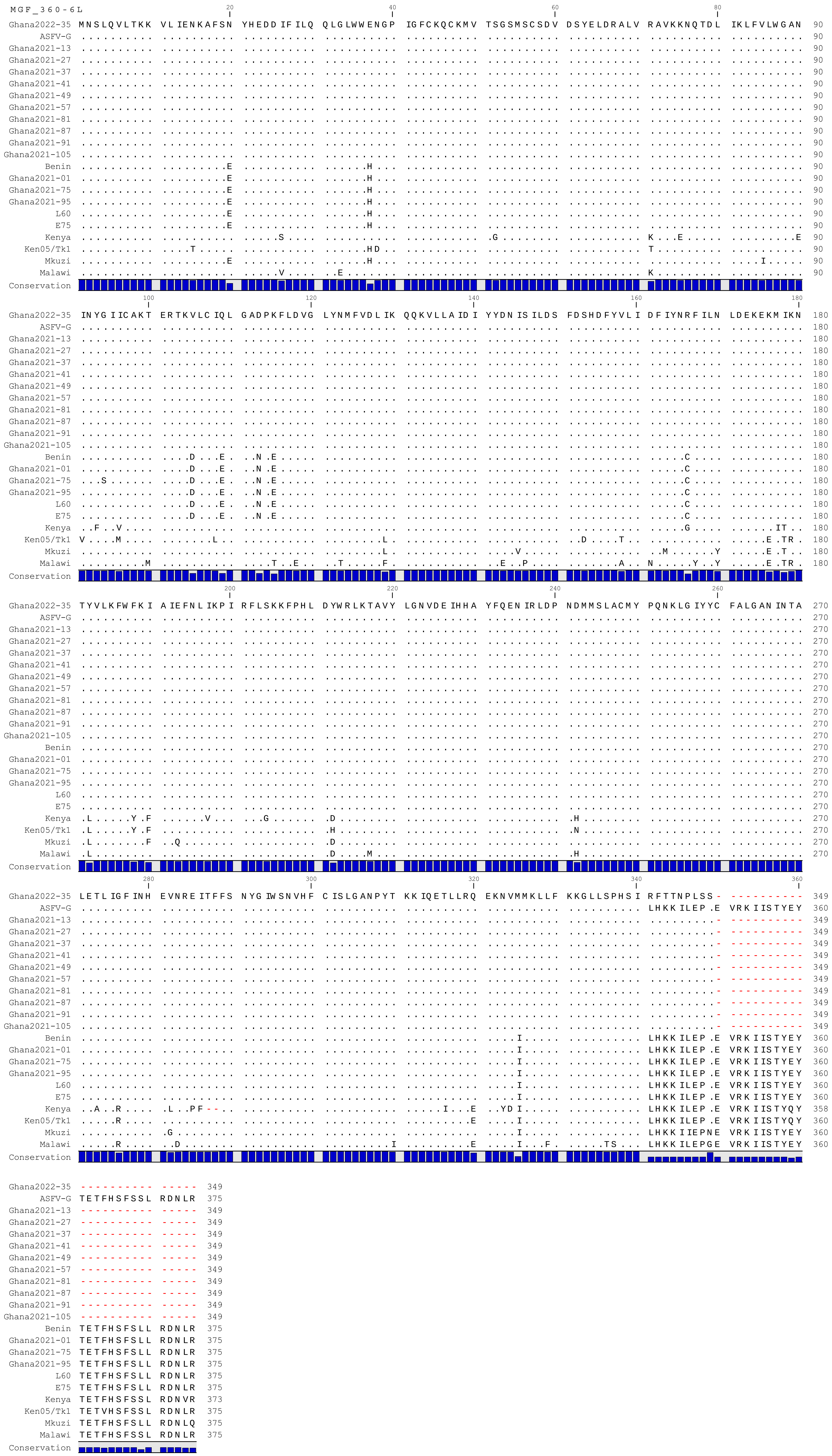
MGF_360-6L

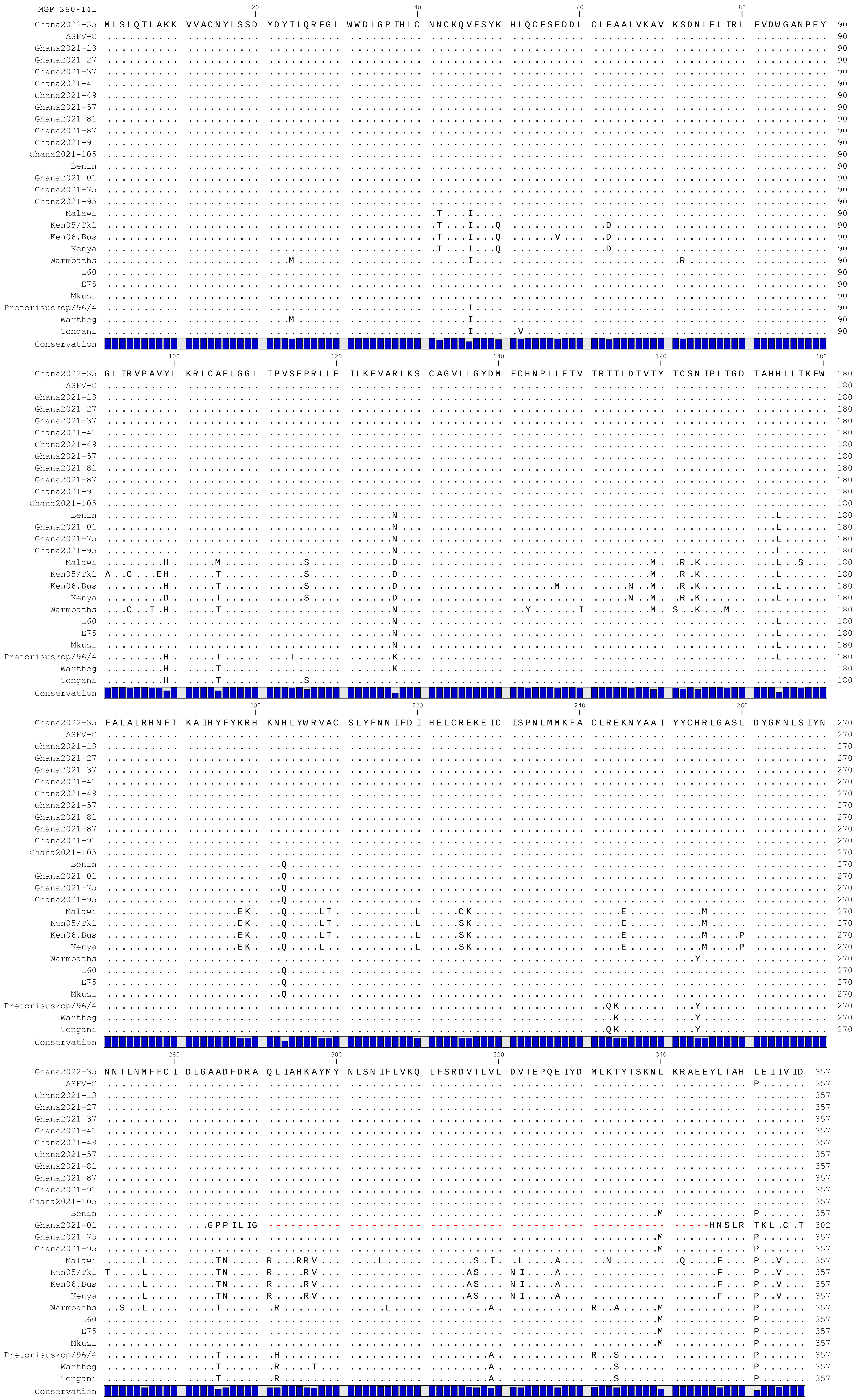
MGF_360-14L
Ghana2022-35 MLSLQTLAKK VVACNYLSSD YDYTLQRFGL WWDLGPIHLC NNCKQVFSYK HLQCFSEDDL CLEAALVKAV KSDNLELIRL FVDWGANPEY 90
Ghana2022-35 GLIRVPAVYL KRLCAELGGL TPVSEPRLLE ILKEVARLKS CAGVLLGYDM FCHNPLLETV TRTTLDTVTY TCSNIPLTGD TAHHLLTKFW 180
Ghana2022-35 FALALRHNFT KAIHYFYKRH KNHLYWRVAC SLYFNNIFDI HELCREKEIC ISPNLMMKFA CLREKNYAAI YYCHRLGASL DYGMNLSIYN 270
Ghana2022-35 NNTLNMFFCI DLGAADFDRA QLIAHKAYMY NLSNIFLVKQ LFSRDVTLVL DVTEPQEIYD MLKTYTSKNL KRAEEYLTAH LEIIVID 357
ASFV-G
Ghana2021-13
Ghana2021-27
Ghana2021-37
Ghana2021-41
Ghana2021-49
Ghana2021-57
Ghana2021-81
Ghana2021-87
Ghana2021-91
Ghana2021-105
Benin
Ghana2021-01
Ghana2021-75
Ghana2021-95
Malawi
Ken05/Tk1
Ken06.Bus
Kenya
Warmbaths
L60
E75
Mkuzi
Pretorisuskop/96/4
Warthog
Tengani
Conservation

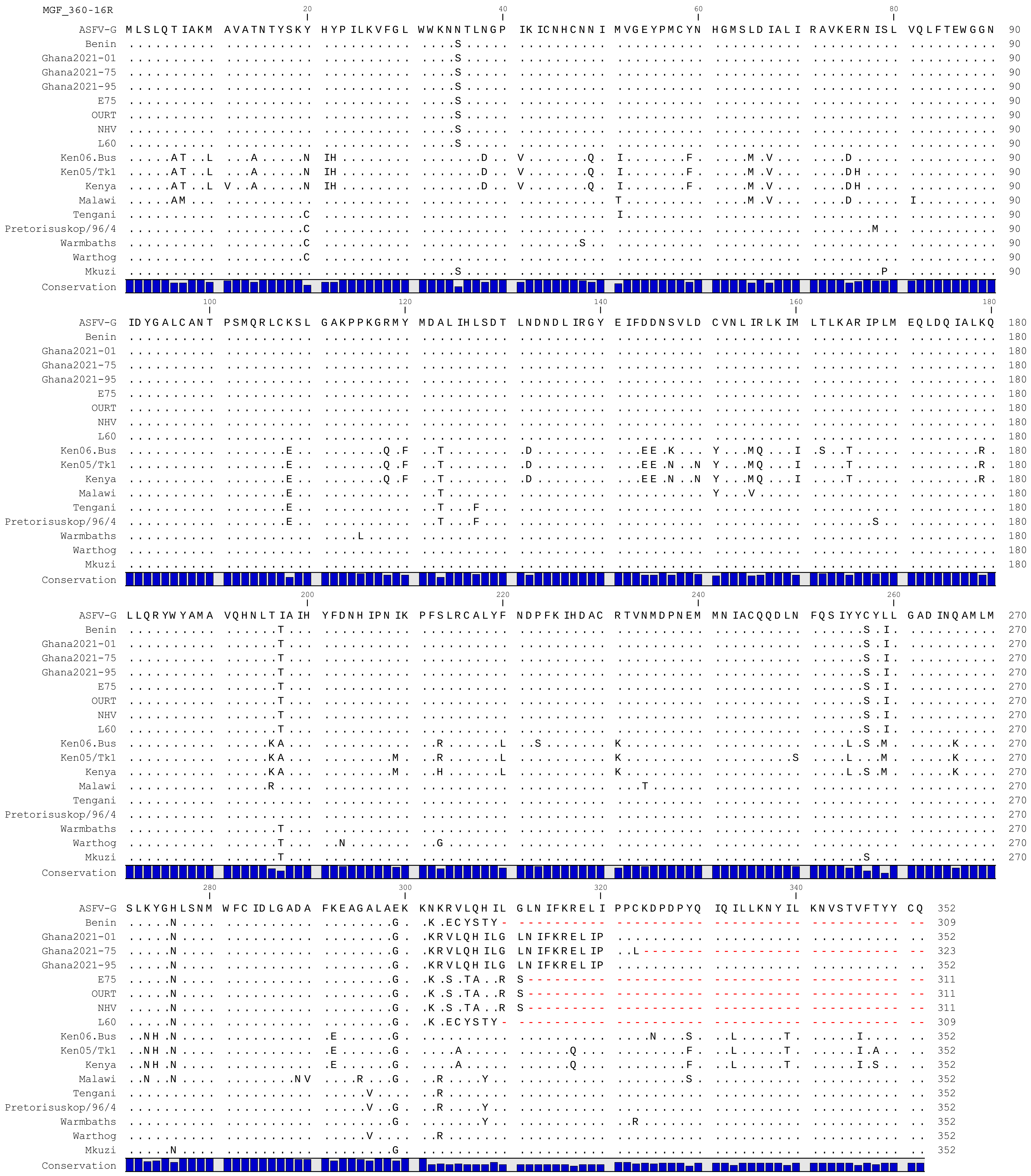
MGF_360-16R
ASFV-G MLSLQTIAKM AVATNTYSKY HYPILKVFGL WWKNNTLNGP IKICNHCNNI MVGEYPMCYN HGMSLDIALI RAVKERNISL VQLFTEWGGN 90
ASFV-G IDYGALCANT PSMQRLCKSL GAKPPKGRMY MDALIHLSDT LNDNDLIRGY EIFDDNSVLD CVNLIRLKIM LTLKARIPLM EQLDQIALKQ 180
ASFV-G LLQRYWYAMA VQHNLTIAIH YFDNHIPNIK PFSLRCALYF NDPFKIHDAC RTVNMDPNEM MNIACQQDLN FQSIYYCYLL GADINQAMLM 270
ASFV-G SLKYGHLSNM WFCIDLGADA FKEAGALAEK KNKRVLQHIL GLNIFKRELI PPCKDPDPYQ IQILLKNYIL KNVSTVFTYY CQ 352
Benin
Ghana2021-01
Ghana2021-75
Ghana2021-95
E75
OURT
NHV
L60
Ken06.Bus
Ken05/Tk1
Kenya
Malawi
Tengani
Pretorisuskop/96/4
Warmbaths
Warthog
Mkuzi
Conservation

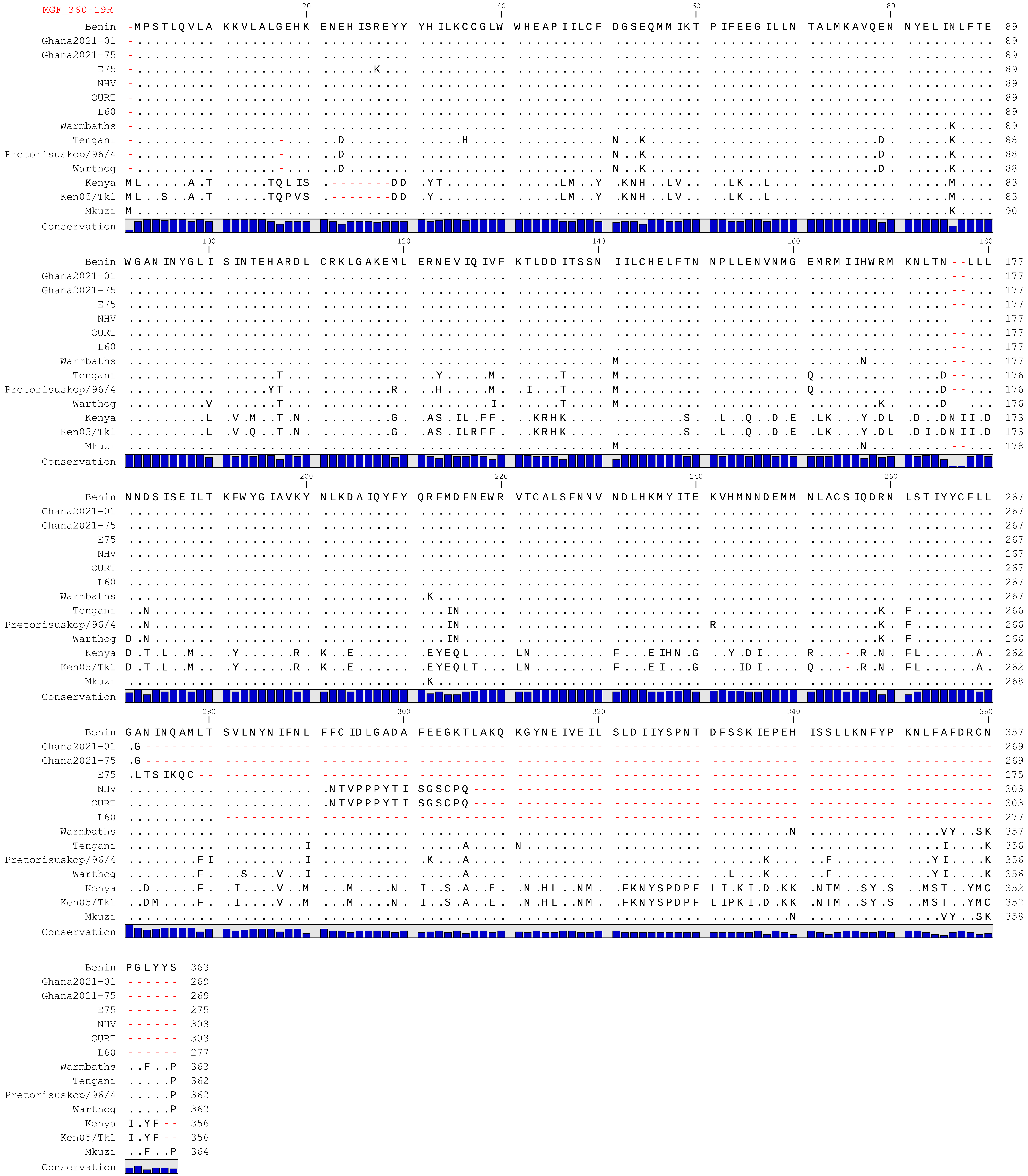
MGF_360-19R
Benin
Ghana2021-01
Ghana2021-75
E75
NHV
OURT
L60
Warmbaths
Tengani
Pretorisuskop/96/4
Warthog
Kenya
Ken05/Tk1
Mkuzi
Conservation

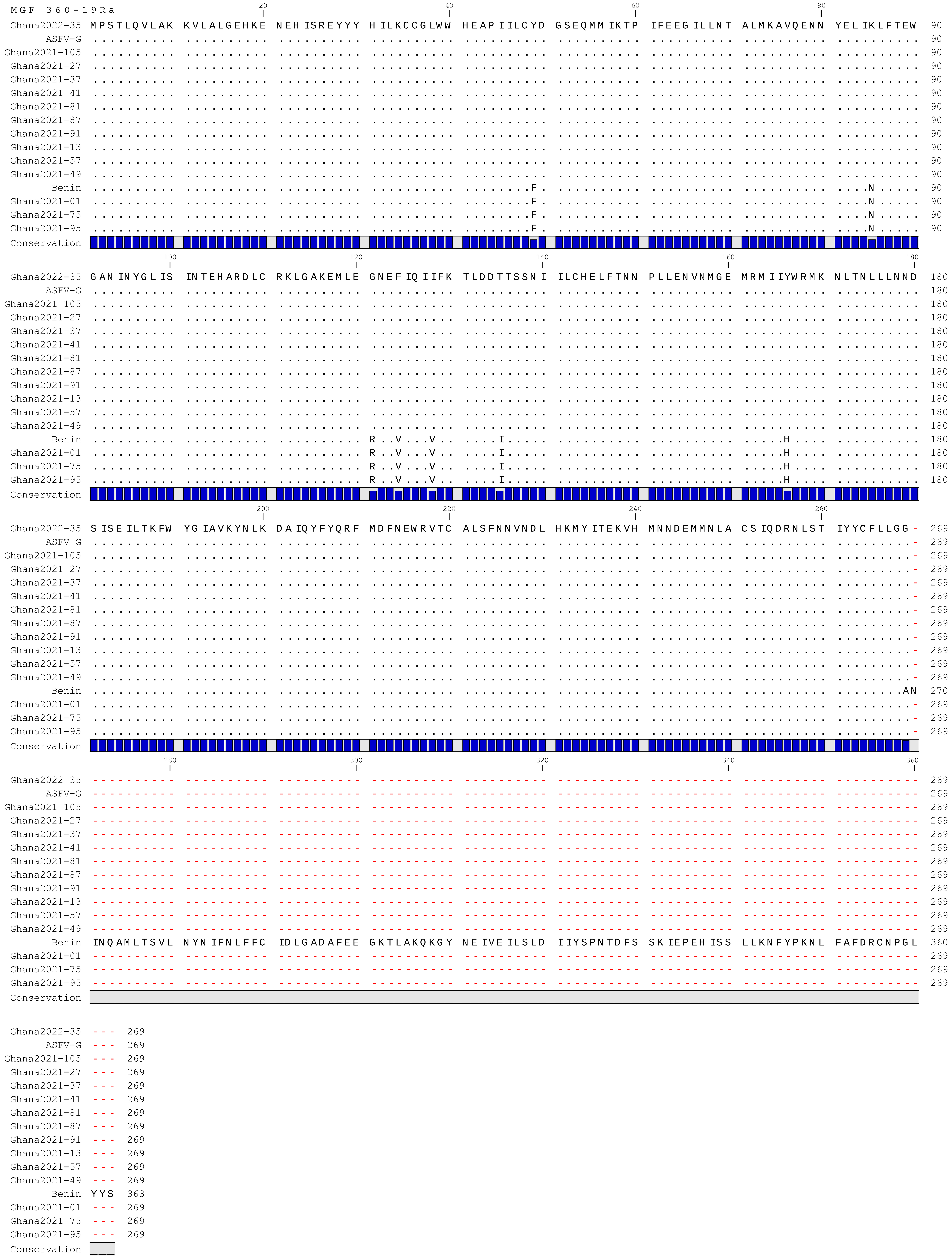
MGF_360-19Ra
Ghana2022-35 MPSTLQVLAK KVLALGEHKE NEHISREYYY HILKCCGLWW HEAPIILCYD GSEQMMIKTP IFEEGILLNT ALMKAVQENN YELIKLFTEW 90
Ghana2022-35 GANINYGLIS INTEHARDLC RKLGAKEMLE GNEFIQIIFK TLDDTTSSNI ILCHELFTNN PLLENVNMGE MRMIIYWRMK NLTNLLLNND 180
Ghana2022-35 SISEILTKFW YGIAVKYNLK DAIQYFYQRF MDFNEWRVTC ALSFNNVNDL HKMYITEKVH MNNDEMMNLA CSIQDRNLST IYYCFLLGG- 269
Benin ...AN 270
Benin INQAMLTSVL NYNIFNLFFC IDLGADAFEE GKTLAKQKGY NEIVEILSLD IIYSPNTDFS SKIEPEHISS LLKNFYPKNL FAFDRCNPGL 360
Benin YYS 363
ASFV-G
Ghana2021-105
Ghana2021-27
Ghana2021-37
Ghana2021-41
Ghana2021-81
Ghana2021-87
Ghana2021-91
Ghana2021-13
Ghana2021-57
Ghana2021-49
Ghana2021-01
Ghana2021-75
Ghana2021-95
Conservation

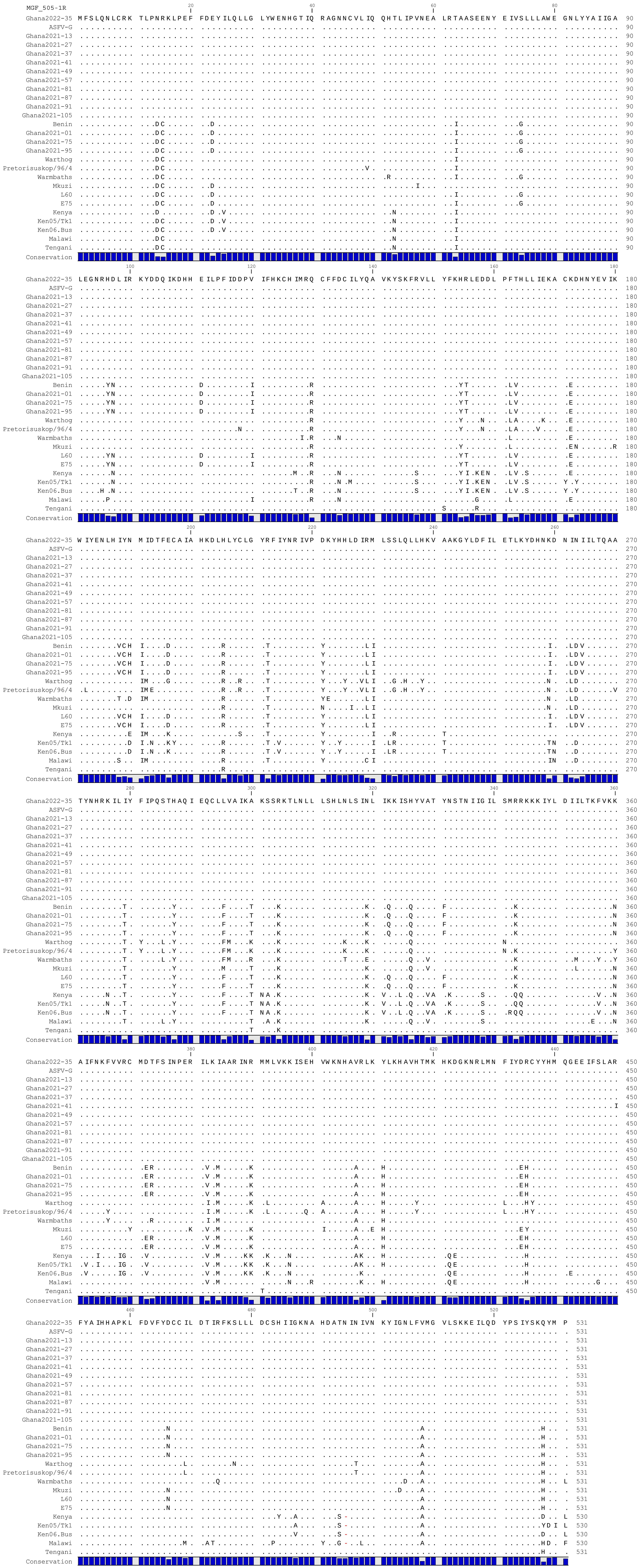
MGF_505-1R
Ghana2022-35
ASFV-G
Ghana2021-13
Ghana2021-27
Ghana2021-37
Ghana2021-41
Ghana2021-49
Ghana2021-57
Ghana2021-81
Ghana2021-87
Ghana2021-91
Ghana2021-105
Benin
Ghana2021-01
Ghana2021-75
Ghana2021-95
Warthog
Pretorisuskop/96/4
Warmbaths
Mkuzi
L60
E75
Kenya
Ken05/Tk1
Ken06.Bus
Malawi
Tengani
Conservation

MGF_505-9R

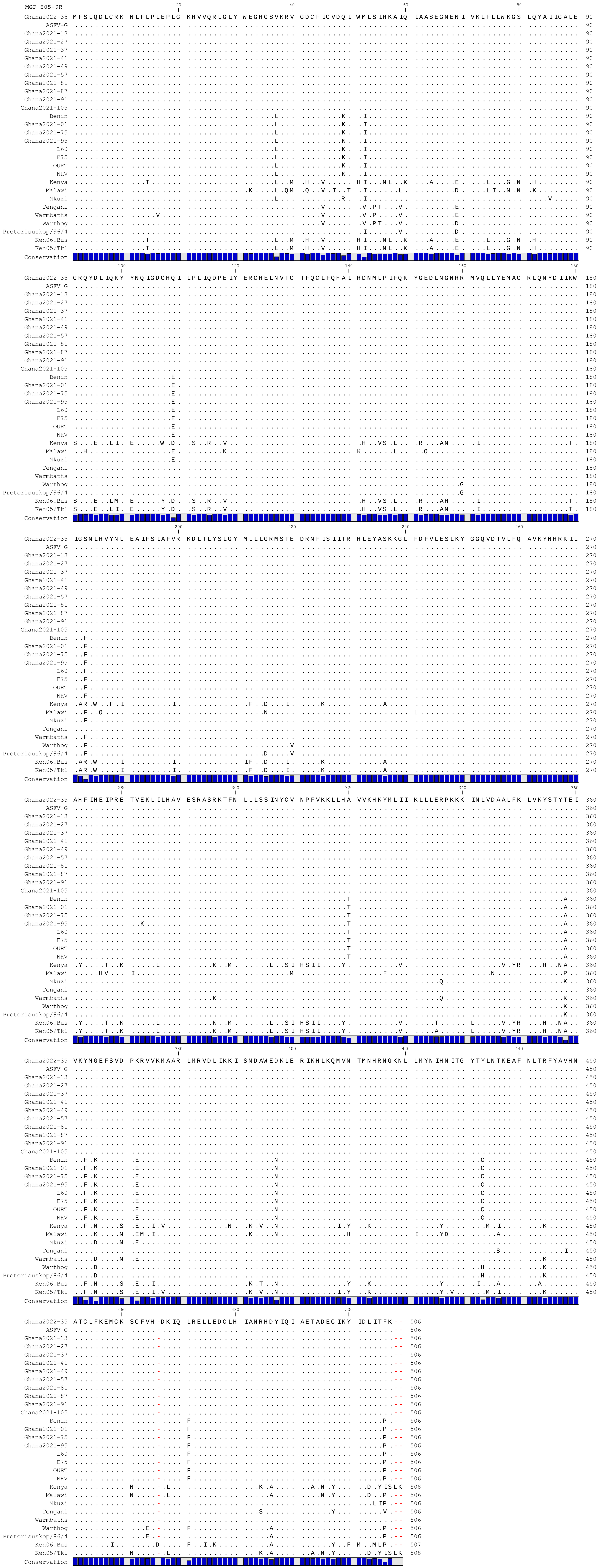

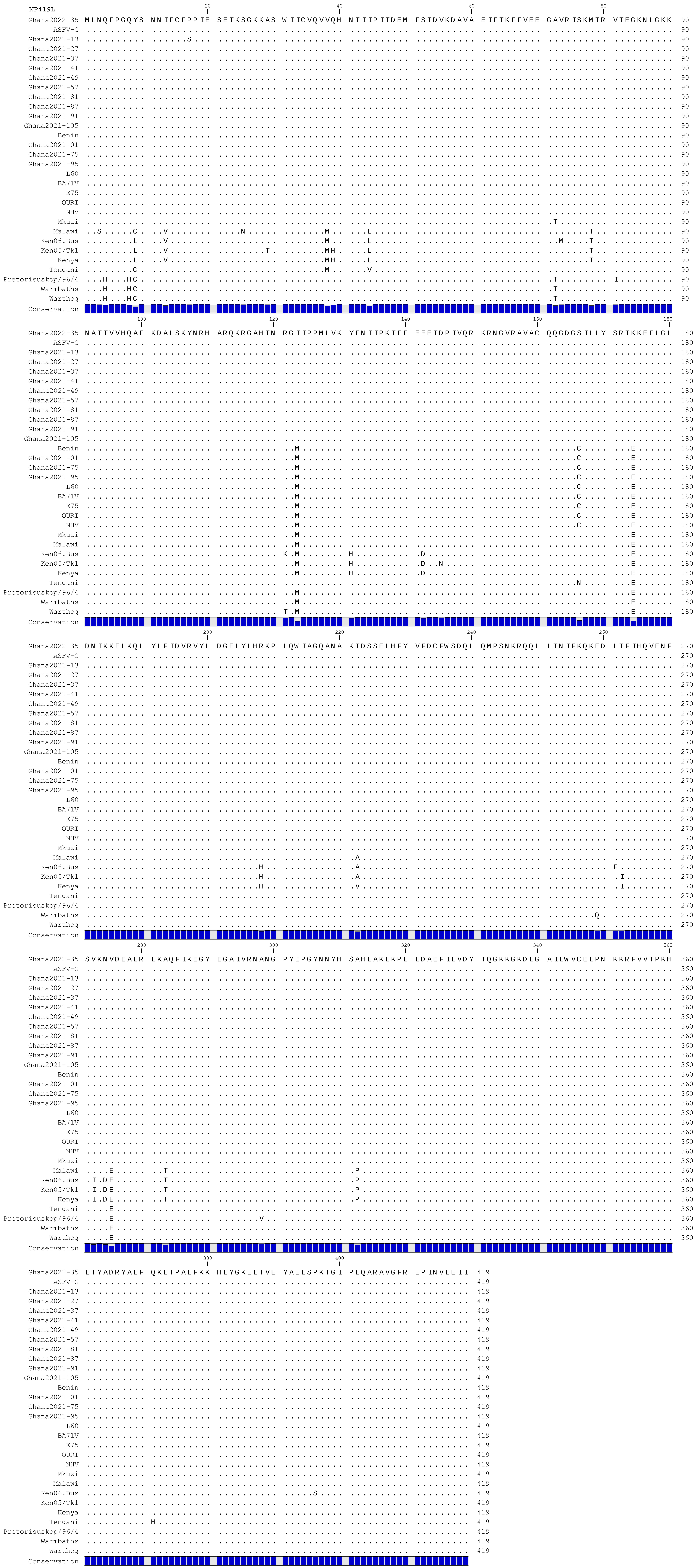
NP419L
Ghana2022-35 MLNQFPGQYS NNIFCFPPIE SETKSGKKAS WIICVQVVQH NTIIPITDEM FSTDVKDAVA EIFTKFFVEE GAVRISKMTR VTEGKNLGKK 90
NATTVVHQAF KDALSKYNRH ARQKRGAHTN RGIIPPMLVK YFNIIPKTFF EETDPIVQR KRNGVRAVAC QQGDGSILLY SRTKKEFLGL 180
DNIKKELKQL YLFIDVRVYL DGELYLHRKP LQWIAGQANA KTDSSELHFY VFDCFWSDQL QMPSNKRQQL LTNIFKQKED LTFIHQVENF 270
SVKNVDEALR LKAQFIKEGY EGAIVRNANG PYEPGYNNYH SAHLAKLKPL LDAEFILVDY TQGKKGKDLG AILWVCELPN KKRFVVTPKH 360
LTYADRYALF QKLTPALFKK HLYGKELTVE YAELSPKTGI PLQARAVGFR EPINVLEII 419
ASFV-G
Ghana2021-13
Ghana2021-27
Ghana2021-37
Ghana2021-41
Ghana2021-49
Ghana2021-57
Ghana2021-81
Ghana2021-87
Ghana2021-91
Ghana2021-105
Benin
Ghana2021-01
Ghana2021-75
Ghana2021-95
L60
BA71V
E75
OURT
NHV
Mkuzi
Malawi
Ken06.Bus
Ken05/Tk1
Kenya
Tengani
Pretorisuskop/96/4
Warmbaths
Warthog
Conservation

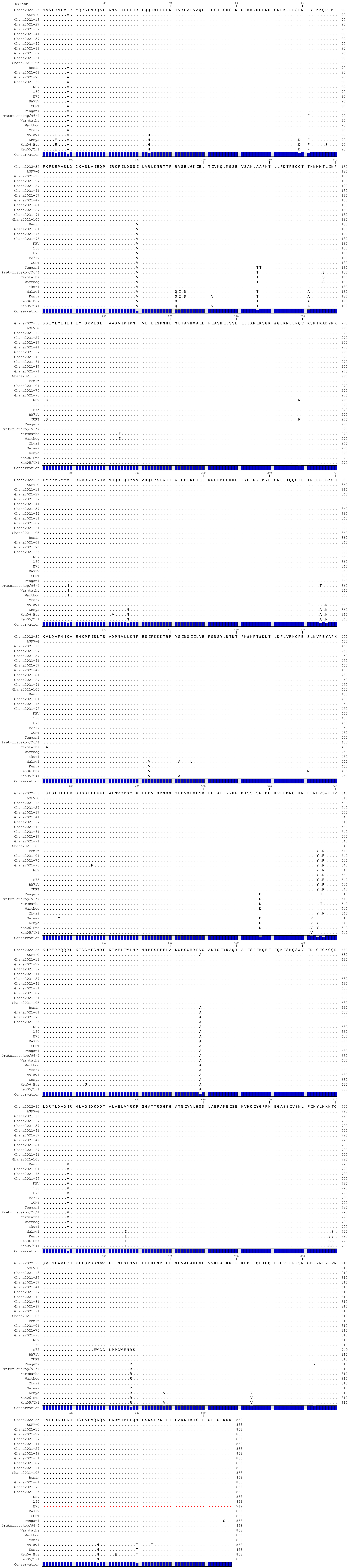
NP868R

NP1450L

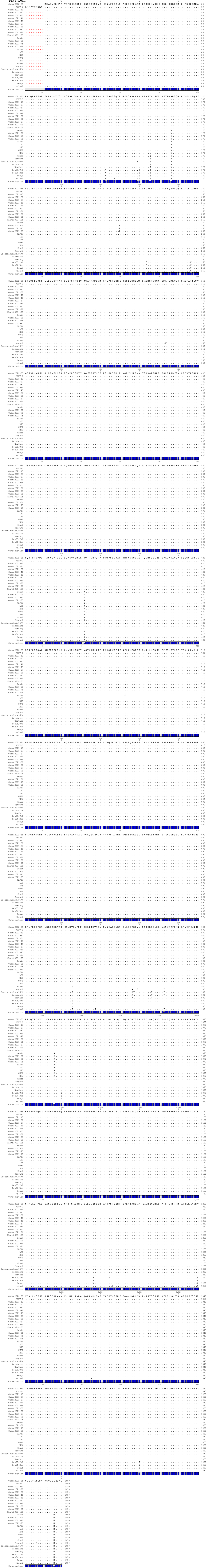

P1192R

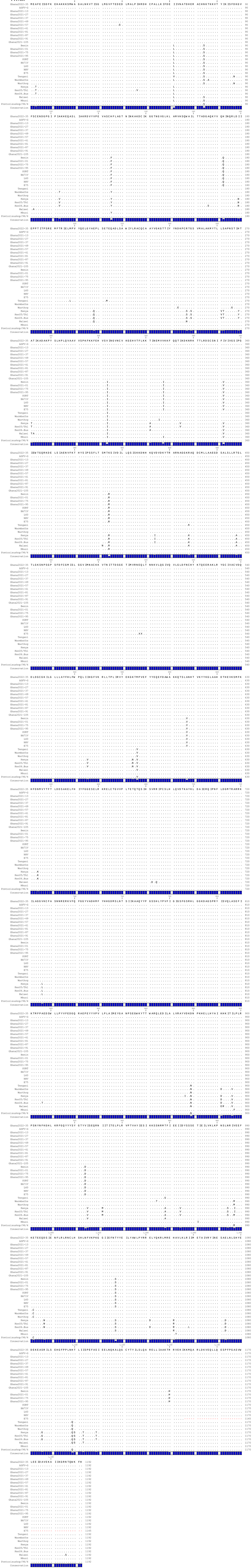

Supplement: Supplementary file 1 [file viruses-16-01293-s001.zip › Supplemental Figure S1.pdf]
